# Spontaneous Reaction of Oleacein and Oleocanthal with Primary Amines: A Biochemical Perspective

**DOI:** 10.3390/molecules30071645

**Published:** 2025-04-07

**Authors:** Daniel Di Risola, Davide Laurenti, Francesca Ferraro, Alessia Ciogli, Simone Manetto, Yuri Gazzilli, Rodolfo Federico, Antonio Francioso, Luciana Mosca, Roberto Mattioli

**Affiliations:** 1 Department of Biochemical Sciences “A. Rossi Fanelli”, Sapienza University of Rome, pl.e Aldo Moro 5, 00185 Rome, Italy; daniel.dirisola@uniroma1.it (D.D.R.); davide.laurenti@uniroma1.it (D.L.); francescaferraro998@gmail.com (F.F.); 2 Department of Chemistry and Technology of Drugs, Sapienza University of Rome, pl.e Aldo Moro 5, 00185 Rome, Italy; alessia.ciogli@uniroma1.it (A.C.); simone.manetto@uniroma1.it (S.M.); yuri.gazzilli@uniroma1.it (Y.G.); 3 Active-Italia S.r.l., Via delle Terme Deciane 10, 00153 Rome, Italy; federico@active-italia.com; 4 Department of Bioscience and Technology for Food Agriculture and Environment, University of Teramo, 64100 Teramo, Italy; afrancioso@unite.it

**Keywords:** oleacein, oleocanthal, oleo-tris, olea-tris, adduct stability

## Abstract

Oleacein (Olea) and Oleocanthal (Oleo) are two phenolic compounds found in olive oil. Cell and animal studies have shown these two compounds can modulate inflammation, cancer, and neurodegenerative diseases. Unfortunately, the study of the pharmacokinetics of these two compounds appears difficult due to their high reactivity with primary amines. Indeed, the presence of primary amines in culture media and biological fluids raises the question as to whether the observed biological effects are attributable to the parent compounds or to their amine derivatives. In the present work, we investigated the adduct formation between Olea or Oleo and tris(hydroxymethyl)aminomethane (Tris), a well-known primary amine used primarily as a buffer system, showing that the reaction kinetics were extremely rapid. In addition, we assessed whether the newly formed Tris adducts, i.e., Olea-Tris and Oleo-Tris, retained their antioxidant capacity by means of the ABTS and DPPH radical scavenging assays, showing that their activity was partially maintained. Finally, we evaluated the anti-inflammatory activity of these adducts on murine BV-2 microglial cells stimulated with lipopolysaccharide (LPS) and kept in an amine-free culture medium, showing how the biological response varied as the compound was degraded. Taken together, these data demonstrate that the biological effects reported in the literature are mainly due to the amino-derivatives of Olea and Oleo rather than the polyphenols derived from their breakdown (tyrosol and hydroxytyrosol).

## 1. Introduction

Virgin olive oil is a basic ingredient of the Mediterranean diet and part of the food culture of the surrounding countries. Its highest edible quality is extra virgin olive oil, which, according to the relevant International Olive Council, is defined as “virgin olive oil which has a free acidity, expressed as oleic acid, of not more than 0.80 g per 100 g and the other physico-chemical and organoleptic characteristics of which correspond to those fixed for this category in this standard”.

The manufacturing process preserves minor components from degradation and confers a high content of polyphenols to olive oil, a class of very interesting compounds characterized by a broad spectrum of activities, enabling them to modulate many physiological functions via their antioxidant and anti-inflammatory activity [1,2,3]. Approximately 30 to 40 different polyphenols are present in olive oil [4], but the most represented and studied are those derived from ligstroside and oleuropein [5,6], i.e., oleocanthal (Oleo) and oleacein (Olea), respectively [7].

Ligstroside and oleuropein, present in olive tree and drupes, are produced by the condensation of their precursors, tyrosol (Ty) or hydroxytyrosol (HTy), respectively, with intermediates produced by the secoiridoid biosynthesis pathways, forming the glycosylated form of ligstroside and oleuropein. During the manufacturing process of olive oil, olives are crushed, thereby liberating enzymes, which catalyze the deglycosylation and demethylation of ligstroside and oleuropein, ultimately generating the dialdehyde forms Oleo and Olea, respectively [8].

Olea and Oleo belong to the class of secoiridoids, a group of compounds found in all Oleaceae plants, including the European olive tree (*Olea europaea* L.). In recent years there has been a great interest in utilizing these molecules for biomedical applications to target inflammatory and neurodegenerative diseases and cancer [9,10,11]. Several studies indicate that olive oil polyphenols can counteract cardiovascular diseases through their anti-inflammatory and anti-hypertensive activity [12] and hamper Alzheimer’s disease neurodegenerative processes via the inhibition of tau and amyloid beta aggregation and via the inhibition of neuroinflammation [13].

One of the major drawbacks that hampers the wide clinical use of olive oil secoiridoids is their poor stability and low bioavailability. Quite surprisingly, Oleo and Olea rapidly disappear when dissolved in biological fluids or in tissue culture media [14]. Researchers have focused on the pharmacokinetics of these molecules, discovering that once injected in the tail vein of mice, Oleo quickly disappeared from plasma within the first few minutes and became undetectable [14,15,16,17]. The same phenomenon was evidenced when Oleo was dissolved in tissue culture media such as DMEM/F12, and this behaviour was attributed to the rapid condensation of the dialdehyde groups of Oleo with primary amines present in those fluids, which give rise to an unusual tetrahydropyridinium [14]. It can be hypothesized that the same reactivity applies to Olea, since it differs from Oleo only because of an extra hydroxyl group on the phenol ring and it possesses the same two reactive aldehyde groups.

By exploiting the high reactivity of aldehyde groups, it has already been demonstrated that the conversion of Olea and Oleo into sulfonated adducts preserves their COX-inhibitory properties [18]. Theoretically, Oleo and Olea can form Schiff bases with any given primary amine present in solution, from amino acids to peptides, to polyamines, to biogenic amines, potentially giving rise to a plethora of different compounds. The formation of such adducts is expected to be rapid [14]; therefore, it cannot be excluded that the biological activity of these two polyphenols could be attributed to their adducts and not to the intact molecules. To test this hypothesis, we designed a set of in vitro experiments aimed at examining the following: (a) whether Olea has the same reactivity as Oleo towards primary amines; (b) whether Oleo and Olea can form adducts with tris(hydroxymethyl)-aminomethane (Tris), an amine not present in biological media and tissues; and (c) whether the Oleo and Olea adducts formed with Tris exert biological activity comparable to that of the parent molecules.

To this purpose, we conducted a series of experiments aimed at verifying the formation of the adducts via chromatographic and spectrometric measurements (LC/MS, HRMS, and NMR) and in order to study their stability in solution. Further to this, we specifically synthesized Oleo and Olea adducts with Tris, and we tested their antioxidant and anti-inflammatory activity in vitro and in cell cultures, comparing the results with those obtained by utilizing the pure Oleo and Olea.

## 2. Results

### 2.1. Adduct Formation and Structural Characterization

**Figure 1:**
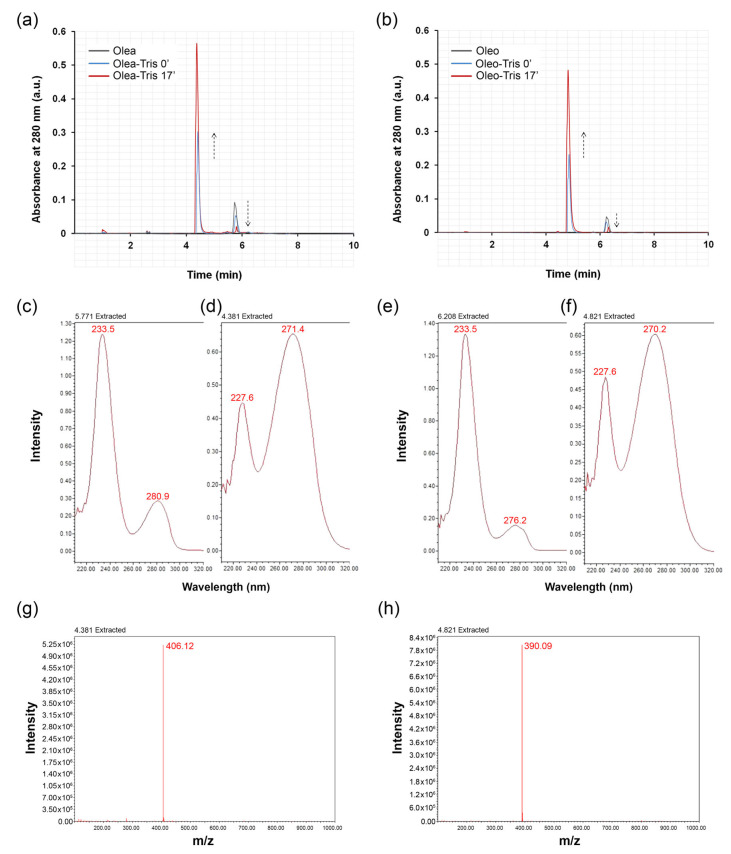
Chromatographic analysis of Olea, Oleo, and their respective adducts. (**a**) A total of 1 mM Olea was diluted in water, mixed with Tris HCl (1:100), and analyzed via UHPLC-DAD-MS at different timepoints (dark gray line: Olea; blue line: Olea + Tris at time 0; red line: Olea + Tris after 17 min of incubation). (**b**) A total of 1 mM Oleo was diluted in water, mixed with Tris (1:100), and analyzed via UHPLC-DAD-MS at different timepoints (dark gray line: Oleo; blue line: Oleo + Tris at time 0; red line: Oleo + Tris after 17 min of incubation). (**c**–**f**) UV-VIS spectra of Olea, Olea-Tris, Oleo, and Oleo-Tris, respectively. (**g**,**h**) Mass spectra of Olea-Tris and Oleo-Tris extracted at their respective peaks from total ion current (TIC) trace. In (**a**,**b**), the dotted arrows show the decrease and increase in Olea/Oleo and Olea-Tris/Oleo-Tris, respectively.

To investigate the ability of Olea and Oleo to form adducts with Tris HCl, each of these two secoiridoids was dissolved in water at a concentration of 1 mM and mixed with a 100-fold molar excess of Tris at room temperature. Analysis via UPLC-DAD-MS of the incubates revealed a time-dependent decrease in the Olea or Oleo amount alongside a concomitant increase in a new product, showing a lower retention time (4.38 or 4.82 min, respectively) (Figure 1a,b) and an *m*/*z* of 406.12 or 390.09, respectively (Figure 1g,h), compatible with that of a dehydrated Schiff base adduct between the secoiridoid and the amine compound.

The analysis of the UV-VIS absorption spectra of individual peaks revealed a significant difference between Olea, Olea-Tris, Oleo, and Oleo-Tris (Figure 1c–f). Specifically, Olea exhibited an absorption peak at 280.9 nm (Figure 1c), Olea-Tris displayed a peak at 271.4 nm (Figure 1d), Oleo showed an absorption peak at 276.2 nm (Figure 1e), and finally, Oleo-Tris exhibited a peak at 270.2 nm (Figure 1f).

The kinetics of adduct formation for both Olea and Oleo appeared to be extremely rapid, as the presence of the adduct was detectable even at time zero, corresponding to the technical time required to mix the secoiridoid with Tris and inject the mixture into the column. Within 17 min, the reaction was practically complete, with residual amounts of Olea or Oleo below the quantification limit and a maximum concentration of the adduct. No other compounds were revealed by the chromatographic analysis, indicating that in a short time, the Oleo or Olea were quantitatively transformed into the adducts (Figure 1a,b).

### 2.2. Investigation on the Structure of Olea-/Oleo-Tris Adducts: High-Resolution Mass Spectrometry (HR-MS) and ^1^H-NMR Analyses

**Figure 2:**
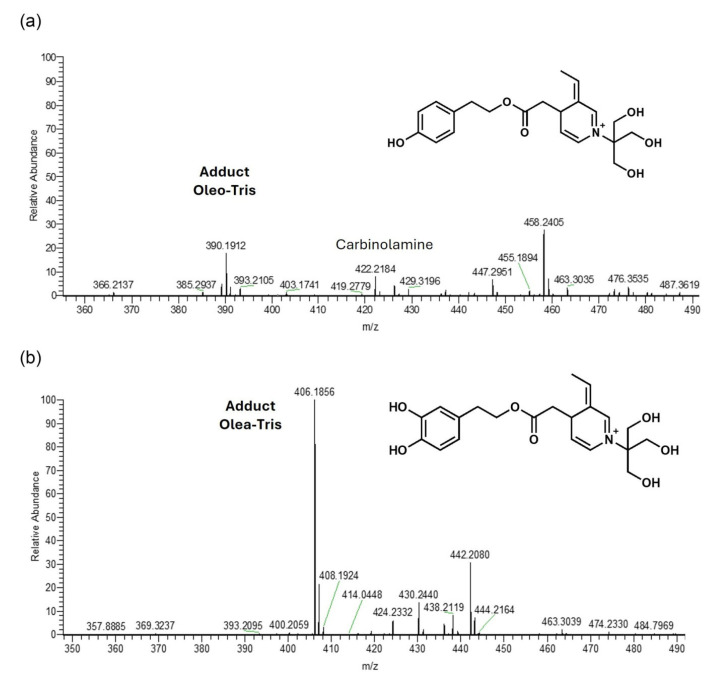
HR-MS flow injection analysis of solutions of Oleo and Tris HCl (**a**) and Olea and Tris HCl (**b**) mixtures. TIC traces were recorded between 350–500 *m*/*z*. The hypothesized structures are shown in the figure.

**Figure 3:**
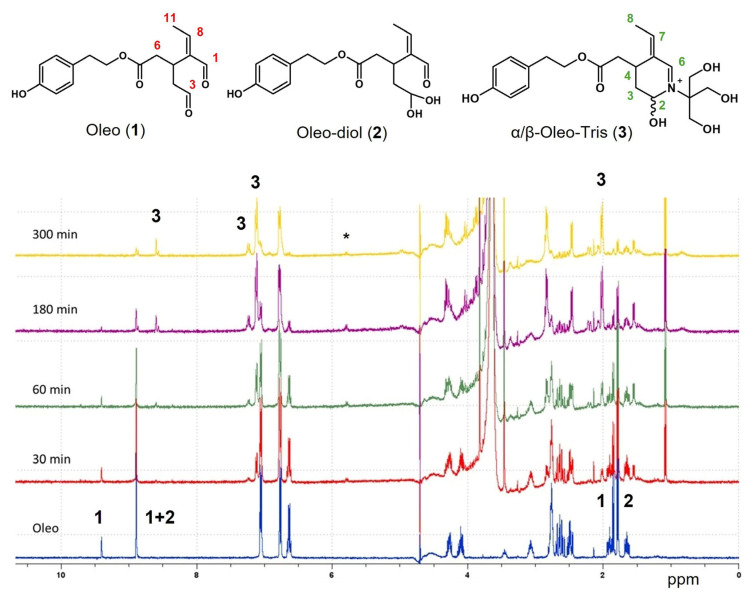
^1^H-NMR of Oleo/Tris HCl mixture in D_2_O over time. Spectra acquired with ws. Numbers in black on the spectra correspond to signals distinctive of the structures shown above in the figure. The asterisk highlights the presence of a new signal not related to **1**–**3** structures.

Aiming to investigate the structure of the secoiridoid–Tris adducts, combined HR-MS, 1D-NMR, and 2D-NMR experiments were employed similarly to a recently published work [14], where the reaction between Oleo and glycine (and other amino acids) was studied. Though HR-MS, two solutions of Oleo and Olea added to Tris HCl (molar ratio 1/2) were prepared in DMSO, diluted in MeOH (dilution 1/1000), and analyzed in the ESI-positive mode. After 1 h of stirring, signals corresponding to the masses already acquired in low resolution were clearly recorded. In detail, ions at 390.1912 *m*/*z* and at 406.1854 *m*/*z*, respectively, were in agreement with the molecular formula of C_21_H_28_NO_6_ ([M]^+^, Δm = 0.32 ppm) and C_21_H_28_NO_7_ ([M]^+^, Δm = 1.48 ppm). The two structures bearing a pyridinium-like scaffold, reported in Figure 2, were hypothesized tentatively at first in order to clarify their structure. Conversely, Darakjian and co-workers identified for oleocanthal and glycine a structure similar to the one in Figure 2a but hydrated (plus 18 a.m.u.) with hydroxyl group in the alpha position to the nitrogen (see Figure 3). In addition, by spectra inspection, ions corresponding to carbinolamine intermediates were also recorded in both systems (Supplementary Materials for zoomed spectra), attesting that a single aldehyde of Oleo and Olea reacts with the amino group of Tris. Moreover, no signals related to the active intermediates achieved for the reaction of both aldehydes with two equivalents of Tris were found.

Moving to the ^1^H-NMR study, the reaction between the dialdehydes and primary amine of Tris was followed over time in D_2_O for both the Oleo/Tris HCl and Olea/Tris HCl mixtures, and all spectra are reported in the Supplementary Materials. The reaction was carried out in the NMR tube at almost 2 mg/mL of secoiridoid and with two equivalents of Tris HCl. When Oleo was dissolved in D_2_O, the diol/monoaldehyde **2** became greater than the native dialdehyde **1**. The doublet at 1.78 ppm (*J* = 7.05 Hz), corresponding to the methyl group (C-11) of **2**, was bigger than the doublet at 1.84 ppm (*J* = 7.05 Hz), its analogue in Oleo, with a 70/30 molar ratio. The C-3 and C-1 aldehydic protons gave two distinct signals at 9.40 ppm and 8.90 ppm, respectively (see blue trace of Figure 3). In the aryl region, in addition to the two doublets of phenol, the quadruplet at 6.64 ppm (*J* = 7.08 Hz) corresponded to the proton at C-8 (Figure 3) that was coupled with methyl C-11 (*J* = 7.04 Hz). This CH_3_-CH spin system was also confirmed by a selective gradient TOCSY experiment (Figure S9). After 30 min of Tris HCl addition, small changes in the aldehydic and the aryl regions of Oleo were detectable, which became clear after 60 min following the start of the reaction. In detail, the signals at 9.41 ppm and at 1.84 ppm noticeably decreased, and at the same time, new signals at 8.65 ppm (s), 7.25 ppm (q), 7.15 ppm (d), and 5.85 ppm (q) appeared, as well as a new broad doublet at 2.01 ppm.

After 180 min, the signal of dialdehyde at 9.41 ppm resulted in a trace in the background, the second aldehydic signal at 8.90 decreased, and two singlets at 8.65 and 8.58 ppm appeared. These two singlets, together with the quadruplet at 7.25 ppm, the doublet at 7.15 ppm, and the more intense doublet at 2.01 ppm, were related to a new compound, a cyclization product of Tris and Oleo (Figure 3, structure **3**). After the reaction of the amino group with the aldehyde C-1, the nitrogen reacted with the second aldehydic group of Oleo, providing the cyclic product as α and β isomers due to the axial or equatorial position of hydroxyl. Two sets of singlets (8.65 and 8.58 ppm) and doublets (2.01 and 2.06 ppm) confirmed the presence of two epimers of the Oleo-Tris adduct **3**. Again, selective gradient TOCSY experimentation confirmed the CH_3_-CH coupling (C-8 and C-7 in structure **3**), and the ^1^J heteronuclear ^1^H-^13^C correlations were obtained by HSQC for proton C-6 (164 ppm) and for proton C-7 (167 ppm), which is in agreement with data from the literature (see Supplementary Materials). The aldehydic signals corresponding to Oleo almost disappeared after 5 h, attesting to its complete transformation, while the residual signal of diol/monoaldehyde **2** disappeared after 24 h (Figures S15 and S16). Notably, after the addition of Tris, the small signal (q) at 5.85 ppm was constantly present in all spectra, and its intensity slowly increased over time. A similar trend was recorded when Olea reacted with Tris HCl (see Supplementary Materials). In both cases, after 24 h, the spectra showed few and broadened signals, attesting to the degradation of the product. From the literature data, some of the signals correlated with the signals of Ty and HTy. Unfortunately, although in line with the results reported by Darakjian and co-workers [14], these results are not in agreement with those obtained by HR-MS. Taking this into account, in the presence of a large amount of Tris, the adduct formation was very fast, and it resulted in an almost stable reaction mixture for 3 h (see the next section, Section 2.3); the Oleo-Tris adduct was freshly prepared in the presence of 100 equivalents of Tris HCl, and after 20 min of reaction time, the corresponding peak of the adduct was isolated by multiple injections in order to collect almost 1.5–2.0 mg of product by the UHPLC system.

**Figure 4:**
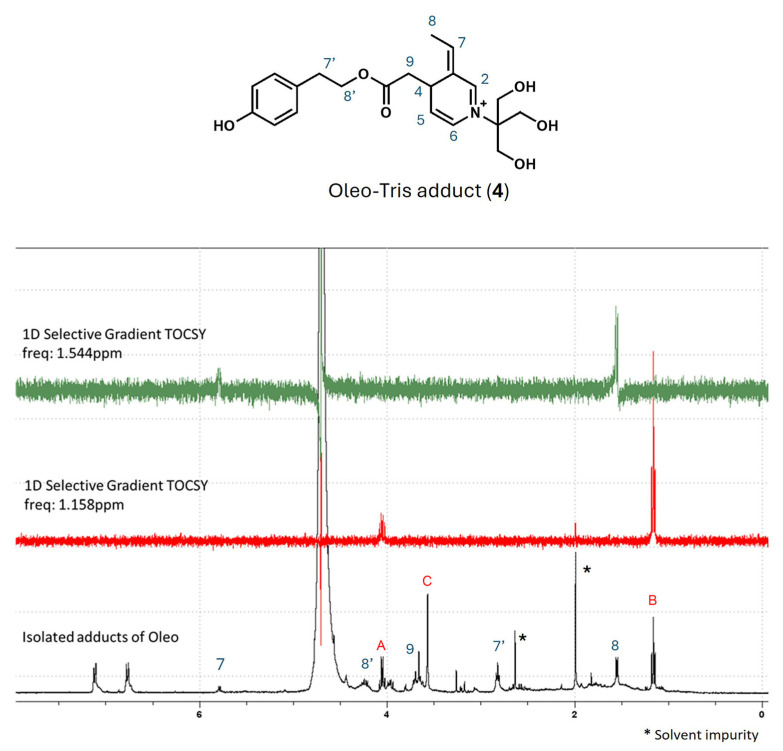
^1^H-NMR spectrum of isolated Oleo-Tris adducts in D_2_O and two 1D selective gradient TOCSY spectra. Signals of solvent impurity are market with asterisk. Unknown A, B and C signals are not related to structure **4** (see main text). **Top**: structures of identified adducts.

The collected fractions were lyophilized and then redissolved in D_2_O to carry out the NMR analysis. Before dissolution in D_2_O, a small amount of the sample was analyzed by MS, recording a 390.09 *m*/*z* signal as the main ion (Figure S38). The ^1^H-NMR spectrum of the isolated adduct showed two signals as broad doublets in the aromatic region, a quadruplet at 5.85 ppm coupled with a doublet at 1.54 ppm (CH_3_-CH), as confirmed by selective gradient TOCSY/2D-COSY experiments (Figure 4). Additional signals, marked in blue, were assigned by 2D-COSY (Figures S34–S36), and a plausible structure is reported in the figure. By the complete inspection of ^1^H-NMR, unknown signals, marked with A, B, and C, showed a 2/3/2 ratio and were in agreement with the CH_2_-CH_3_ system and an isolated CH_2_. Unfortunately, the experimental data did not provide exhaustive information on the possible presence of a constitutional isomer of the identified adduct **4** or of a degradation product. However, the isolation of the intermediate confirmed that the α/β isomers 3 were not present when Tris HCl was employed in a large excess.

### 2.3. Kinetics of Adduct Formation and Their Stability

**Figure 5:**
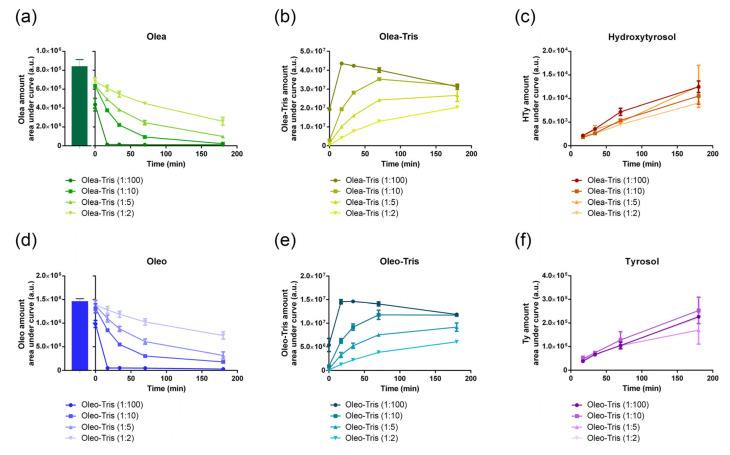
Olea-Tris and Oleo-Tris formation kinetics and their stability during the incubation time. (**a**,**d**) Olea and Oleo amount, expressed as the area under curve and analyzed by UPLC-DAD-MS. The dark green and blue bars represent the initial amount of Olea and Oleo before adding Tris. (**b**,**e**) Formation kinetics of Olea-tris and Oleo-Tris adducts and their stability as a function of time. (**c**,**f**) HTy and Ty formation during the incubation time as a degradation by-product of Olea-Tris and Oleo-Tris. The values are reported as mean ± SD.

Although the rate of adduct formation was rapid, it appeared to depend on the secoiridoid/Tris ratio used (Figure 5). Specifically, at a secoiridoid/Tris molar ratio of 1:100, the Olea and Oleo were fully transformed into adducts within the first 17 min, with the maximum adduct formation observed. However, at lower secoiridoid/Tris ratios (1:10, 1:5, and 1:2), both the reaction rate and the depletion rate of the Olea and Oleo were reduced (Figure 5a,b,d,e).

Additionally, the stability of the adducts appeared to be relatively low. Over a period of 3 h, the amount of adduct formed at 17 min under a 1:100 molar ratio gradually decreased (Figure 5b,e). Concurrently, chromatographic analysis of the incubation mixture revealed that HTy and Ty appeared in the incubation mixture (Figure 5c,f) as degradation products of Olea and Oleo, respectively.

**Figure 6:**
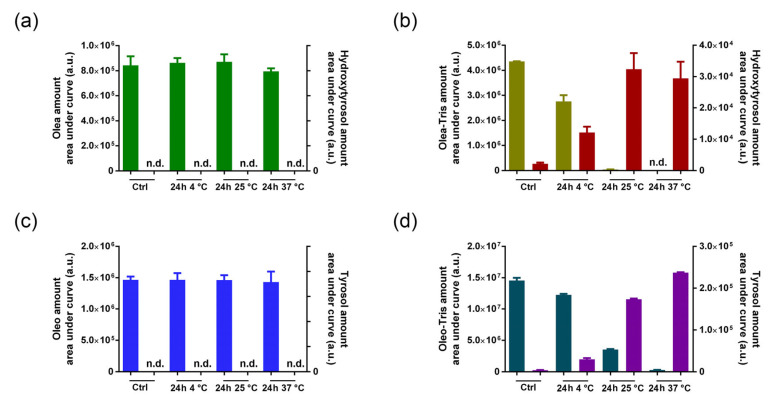
Stability of Olea, Oleo, and their respective adducts in water after 24 h at 4, 25, and 37 °C (**a**) Olea (green) and Hydroxytyrosol. (**b**) Olea-Tris (olive green) and Hydroxytyrosol (red). (**c**) Oleo (blue) and Tyrosol. (**d**) Oleo-Tris (dark green) and Tyrosol (violet). (n.d.: not detected). The values are reported as mean ± SD.

To examine the stability of the adducts in better detail, including the influence of temperature, both the adducts (Olea-Tris and Oleo-Tris at a 1:100 ratio) and their precursors (Olea and Oleo) were dissolved in water and stored at 4, 25, and 37 °C. After 24 h, the residual quantities were measured. While the Olea and Oleo remained stable over the monitoring period regardless of the temperature, both the adducts, Olea-Tris and Oleo-Tris, confirmed their instability (Figure 6). Specifically, after 24 h, the Olea-Tris adduct decreased by approximately 30% from the initial amount when stored at 4 °C and nearly disappeared when stored at 25 or 37 °C (Figure 6b). A similar behavior was observed for the Oleo-Tris adduct in the same experimental conditions, although in this case, the stability was slightly higher at both 4 and 25 °C compared to the Olea-Tris adduct (Figure 6d). As expected, the adduct degradation led to a parallel increase in HTy and Ty, reflecting the trend of adduct degradation. Specifically, while the HTy and Ty amounts were minimal in the incubates stored at 4 °C, they were markedly higher in those stored at 25 and 37 °C (Figure 6b,d).

### 2.4. Stability of Olea, Oleo, and Their Respective Adducts in Cell Culture Medium

**Figure 7:**
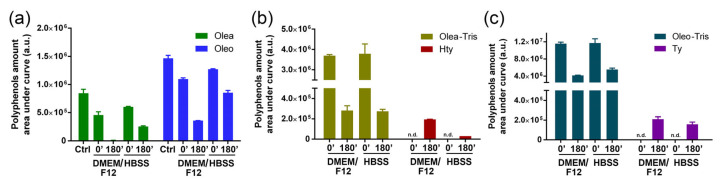
Stability of Olea and Oleo (**a**) and their respective adducts (**b**,**c**) in a cell culture medium (DMEM/F12) and saline buffer (HBSS) at different timepoints. As shown in (**b**,**c**), the appearance of HTy and Ty as degradation by-products was monitored (n.d.: not detected). The values are reported as mean ± SD.

To study the stability of Olea, Oleo, and their respective adducts, time-course measurements were conducted using UPLC-DAD-MS analysis. Specifically, Olea, Oleo, and their respective adducts were dissolved in DMEM/F12 cell culture medium at 37 °C. Their behavior was also tested in HBSS buffer, which, unlike DMEM/F12, lacks amino acids, polyamines, vitamins, or other primary amines. As described in the literature by Darakjian [14], both the Olea and Oleo rapidly disappeared as soon as they were dissolved in DMEM/F12, with their levels already being reduced at time 0 compared to the control and continuing to decrease after 3 h. In the DMEM/F12 medium, during the degradation process, HTy acetate and Ty acetate were formed, as highlighted by Darakjian and colleagues [14]. Conversely, no adducts could be evidenced when Oleo and Olea were dissolved in HBSS, a medium containing only salts and glucose. However, to our surprise, Olea and Oleo showed a scarce stability in this medium, although they were still detectable after 3 h, contrary to what happened in DMEM/F12 (Figure 7a). Moreover, contrary to what was observed in the DMEM/F12 medium, HTy acetate and Ty acetate were not detected in the HBSS medium.

Similarly, the stability of the pre-formed Olea-Tris and Oleo-Tris adducts was tested in both DMEM/F12 and HBSS within 3 h. Similar to Olea and Oleo, their respective adducts also appeared to be unstable, with a decrease in concentration observed in both DMEM/F12 and HBSS (Figure 7b,c). However, unlike Olea or Oleo, there were no significant differences between the residual quantities of each adduct in DMEM/F12 compared to HBSS. As expected, the degradation of Olea-Tris and Oleo-Tris was accompanied by the formation of HTy and Ty, respectively.

### 2.5. In Vitro Antioxidant Activity of Olea, Oleo, and Their Respective Adducts

To investigate the in vitro antioxidant activity of Olea, Oleo, and their respective adducts, DPPH and ABTS assays were conducted at time 0 and after 24 h of incubation in water at 37 °C using ascorbic acid as a reference for scavenging activity. As shown in Table 1, the scavenging activity of Olea, measured both at time 0 and after 24 h, was slightly lower than that of ascorbic acid in both the DPPH and ABTS assays, though within the same order of magnitude. Conversely, no scavenging activity was detected for Oleo in the DPPH assay, although an activity was observed in the ABTS assay. Even so, in the ABTS assay, the scavenging activity of Oleo was lower than that of both Olea and ascorbic acid. No significant differences were observed between time 0 and 24 h.

Regarding the scavenging capacity of the Olea-Tris and Oleo-Tris adducts, the results were more complex. Specifically, the DPPH assay showed good scavenging activity for Olea-Tris at time 0 but not at 24 h, and low or no scavenging activity was observed for Oleo-Tris. Conversely, the ABTS assay showed good scavenging capacity for both Olea-Tris and Oleo-Tris at time 0 but not at 24 h.

### 2.6. Biological Activity of Olea, Oleo, and Their Respective Adducts

To investigate the biological activity of Olea, Oleo, and their respective Tris adducts, gene expression analyses were conducted using real-time PCR for the genes *IL-1β* and *iNOS* in BV-2 cells treated with the pro-inflammatory agent LPS. The choice to focus attention on Tris rather than on other amines capable of forming adducts with Olea and Oleo was driven by the need for clarity and experimental precision. Indeed, in aiming to test these adducts in a cellular system, to determine whether the effects described in the literature arise from Olea and Oleo themselves rather than from their derivatives or degradation products, we selected Tris, as it is not typically present in standard culture media. Using an amino acid or polyamine added to the culture medium would have certainly influenced the cellular response and potentially masked the effect caused by the adducts. Moreover, initially, experiments were conducted in HBSS buffer to eliminate potential confounding elements from primary amines typically present in standard cell culture media, which could react with Olea and Oleo. Subsequently, the results were confirmed in DMEM/F12 medium.

**Figure 8:**
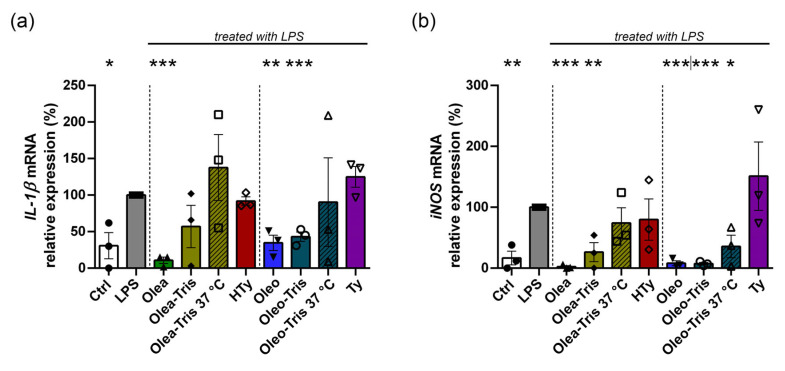
Biological activity of Olea, Oleo, their respective adducts administered as such or degraded at 37 °C for 24 h, HTy, and Ty on BV-2 cells in HBSS medium and treated in the presence or in the absence of LPS for 6 h. The mRNA expression of *IL-1β* (**a**) and *iNOS* (**b**) is shown and was analyzed by real-time PCR. The values were calculated by the 2^−ΔΔCt^ method, normalized with the *RPS27A* housekeeping gene, and compared with LPS treatment (* *p* value < 0.05; ** *p* value < 0.01; *** *p* value < 0.001). The values are reported as mean of percentage ± SEM.

Specifically, BV-2 cells were treated with LPS to induce an inflammatory response and co-treated with the different compounds to test their anti-inflammatory capacity: Olea, Oleo, their respective Tris adducts, the two adducts incubated at 37 °C (degradation-induced), and, finally, HTy and Ty. The gene expression levels of *IL-1β* and *iNOS* in the treated samples were compared to those in control cells not subject to LPS stimulation (Figure 8). The expression of *IL-1β* and *iNOS* significantly increased by approximately 3- and 6-fold, respectively, in the BV-2 cells treated with LPS at 1 µg/mL for 6 h compared to the control cells. Co-treatment with LPS and either Olea or Oleo reduced the gene expression levels, bringing them close to those of the unstimulated control. Similarly, co-treatment with LPS and the adducts also led to a reduction in *IL-1β* and *iNOS* expression, with values similar to those observed in the samples co-treated with LPS and Olea or Oleo. Conversely, neither the adducts degraded at 37 °C for 24 h nor the degradation products, HTy and Ty, significantly reduced *IL-1β* and *iNOS* expression in the LPS-induced samples.

**Figure 9:**
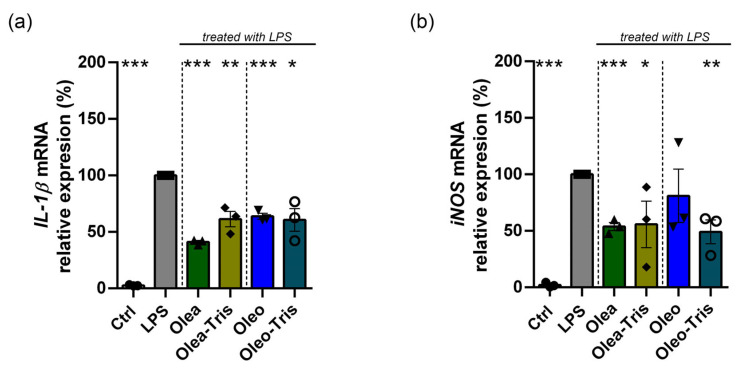
Biological activity of Olea, Oleo, and their respective adducts on BV-2 cells in DMEM/F12 medium and treated in the presence or in the absence of LPS for 24 h. The mRNA expression of *IL-1β* (**a**) and *iNOS* (**b**) is shown and was analyzed by real-time PCR. The values were calculated by the 2^−ΔΔCt^ method, normalized with the *RPS27A* housekeeping gene, and compared with LPS treatment (* *p* value < 0.05; ** *p* value < 0.01; *** *p* value < 0.001). The values are reported as mean of percentage ± SEM.

Finally, although to a lesser extent, similar results were obtained when performing similar experiments using DMEM/F12 medium instead of HBSS (Figure 9).

Taken together, these data suggest that while Olea, Oleo, and their intact adducts exhibited similar anti-inflammatory and antioxidant activities, their degradation products did not demonstrate the same effect.

## 3. Discussion

Olive oil secoiridoids are notable bioactive compounds with significant nutraceutical potential in terms of the prevention and management of cancer, neurodegenerative diseases, and metabolic disorders [5].

The difficulty in detecting these molecules within biological matrices presents a significant challenge in pharmacokinetic studies. This hinders our ability to determine whether the observed biological effects are attributable to the parent compounds themselves or to their metabolites formed in the gastrointestinal tract or plasma [15,16]. Olea and Oleo have a fairly unstable chemical nature, and their detection in biological tissues is therefore also very complicated [17,19]. Kano and colleagues showed that Olea, whether administered *per os* or intravenously, was not detected at all, but its derivatives such as HTy, homovanillic acid, and homovanillyl alcohol (HVA) could be measured [20]. Yerena et al., on the other hand, showed that a large part of Oleo was already metabolized to Ty within the first hour in rat stomach [15]. Other similar evidence is zealously summarized in this review [19].

Olea and Oleo appeared stable when incubated for 4 h at pH 2 at 37 °C [21] but were rapidly degraded in vivo in the gastric environment [16]. As described by Soler et al. [22], simulated gastric digestion in vitro (pepsin/HCl digestion) does not appreciably impair the concentration of Oleo and Olea, whereas duodenal digestion in vitro (pancreatin/bile salts) brings these two polyphenols to non-detectable levels, while Ty and HTy values increase significantly. For the first time, Darakjian and colleagues attempted to shed light on the issue by demonstrating that Oleo is capable of forming adducts with molecules containing primary amines, such as the amino acid glycine [14]. With this work, we validated the findings of Darakjian, demonstrating that adducts of Oleo and Olea can also be formed with non-physiological amines such as Tris, and we aimed to investigate the biological impact of the adducts formed compared to Olea and Oleo themselves, as well as in relation to their degradation products, HTy and Ty. In our experimental setting, Tris was chosen in order to avoid any possible interference of the excess non-reacted reagent in biological media such as tissue culture medium. Tris was not present in the cell culture media, contrary to other amino acids or amines, whose impact on cells could have added a confounding factor.

The structural characterization of the formed adducts confirmed that the Schiff base formed was rearranged into a tetrahydropyridinium product. The kinetics of the reaction depended upon the amount of amine present in the solution and the secoiridoid/Tris ratio. The degradation of the adducts caused the formation of Ty or HTy, which have already been extensively described as degradation products of Oleo and Olea, respectively [23]. A similar pattern of degradation was observed in all the media tested, i.e., water, HBSS, and DMEM/F12, although with significant differences. Indeed, our data indicated that Olea and Oleo were more stable in HBSS buffer than in DMEM/F12 medium. Stability analyses, conducted using UPLC-DAD-MS, revealed that during the degradation process in DMEM/F12 medium, HTy acetate and Ty acetate were formed, corroborating the findings of Darakjian and colleagues [14]. Interestingly, HTy acetate and Ty acetate were not produced during the degradation of the molecules in water or HBSS, suggesting that a component present exclusively in DMEM/F12 medium was responsible for the formation of these degradation products.

The evaluation of adduct stability was performed in these three different solutions for the following reasons. In water, there is a total absence of any possible interfering compound on the process. Stability in DMEM/F12 was determined in order to correlate the biological effects observed in cells with the amount of product present at any given time in the solution. HBSS was chosen for specific cell culture experiments to unequivocally assess the effects of Oleo and Olea on cells. Indeed, HBSS is a balanced salt solution containing glucose, in which cells can survive, even if for limited time periods, i.e., a few hours. HBSS does not contain amino acids or amines, which could rapidly react with the secoiridoids. Hence, in this medium, the biological effects observed in the cells must be directly attributed to Oleo or Olea. This enabled us to compare the effects observed with the corresponding adducts without the potential interference of other possible further amine adducts formed when Oleo and Olea were added to the DMEM/F12.

In our work, we showed that under the conditions of the absence of free amines in the culture medium, such as in HBSS, Olea had a much greater effect on *IL-1β* and *iNOS* transcripts than the adduct with Tris, while the effects of Oleo and Oleo-Tris were very similar. What we can see, however, is that when the adducts with Tris were degraded at 37 °C, they had no effect on these transcripts, similar to the effect recorded by cells treated with the hydrolysis products of Olea and Oleo (i.e., HTy and Ty). We would like to point out that the aim of this experiment was to evaluate whether adducts with Tris still possess biological activity, compared to their parent molecules. The effects of Ty and HTy are known in the literature and most likely produce a positive effect on cells and organisms in a different way than the modulation of the transcripts we considered in this work. Another point to consider is that although the medium used was amine-free, we cannot exclude that there were amine compounds in and on the surface of the cells that could have interacted with the Oleo and Olea, and we cannot exclude a possible role for these amines in facilitating the entry of the secoiridoids into the cells.

It is noteworthy that although Olea- and Oleo-Tris are unstable compounds, they are nonetheless able to exert antioxidant activity in vitro and anti-inflammatory effect on cells. In a very interesting way, Iacono and colleagues [24] tested some Oleo synthetic derivatives in LPS-stimulated chondrocytes. Among these derivatives, although derivative 127 did not have a phenolic ring, it was nevertheless able to reduce nitrite production in the same way as its parent molecule. In contrast, derivative 166, which had two alcohol groups instead of aldehyde groups, produced a mild effect on nitrite production, and only at high concentrations. These data are in line with what we saw in this experiment, and it is very likely that aldehyde groups are essential for binding primary amines and producing biological effects. However, the aromatic rings of these polyphenols have also proven to be very potent in terms of neurodegeneration in vitro; in fact, Romanucci et al. [25] studied the interaction between Ty, HTy, and HVA and amyloid beta 1-40 in depth, showing how HTy inhibits aggregation while Ty and HVA stimulate amyloid aggregation. On the other hand, Li et al. [26] described how Oleo is able to react with amino groups of the Tau protein, inhibiting the fibrillation of this protein. Hence, administering secoiridoids in neurodegenerative diseases could make sense, as Olea and Oleo can create Schiff bases with the Tau protein, while the polyphenols from their hydrolysis are able to inhibit amyloid beta fibrillation. However, it has to be taken into account that when Olea and Oleo are combined with primary amines, the aldehyde groups are blocked, and the capacity to interfere with Tau proteins could be lost.

The phenomenon we face here is quite interesting: Olea and Oleo show a biological effect whether they are administered either as such in HBSS or in the form of adducts with a primary amine, such as Tris, which per se does not possesses any known biological activity. The experiment performed in DMEM/F12 confirmed that the biological activity of the possible adducts that may arise in the cell culture medium (including adducts with free amino acids, polyamines, and other biomolecules) was comparable to that of the Tris adducts. On the other hand, it can be noticed that the Olea and Oleo response (−90% approx.) in HBSS for *iNOS* was greater than that seen in DMEM/F12 (<50% approx.). A similar phenomenon was observed for *IL-1β*. Although the two experiments were different in terms of experimental designs (due to the differences between the two culture media), the results clearly indicate that the actual biological effects of Olea and Oleo most likely vary enormously in the literature due to the different reactivity of their aldehyde groups in different media. Paradoxically, studies on DPPH and ABTS radicals show the other side of the coin. In fact, Oleo-Tris seems to be much better at scavenging these two radicals than the parent molecule Oleo. Of note is the fact that Tris was used as a ’neutral’ molecule to block the two aldehyde groups, so the possible binding of these two polyphenols to various biologically active amines could lead to the generation of an array of promising molecules for the treatment of inflammatory diseases.

In general, our data are in line with the most recent literature data on Olea and Oleo. Muñoz-Garcia et al. [27] demonstrated how Olea could negatively modulate the expression of the pro-inflammatory cytokines *IL-1β*, *TNF-α*, *IL-6*, and others while simultaneously lowering the expression levels of *iNOS* (and concomitant nitrite production) in splenocytes stimulated with LPS for 18 h. Similar data were also seen in macrophage cells [28]. Similarly, Gutiérrez-Miranda et al. [29] showed how Olea treatment was able to negatively modulate *TNF-α*, *Il-1β*, and ROS production in BV-2 microglial cells after 4 and 24 h of LPS treatment. Most interestingly, Olea produced similar effects in a murine autoimmune encephalomyelitis model. Wakasugi et al. [30] showed improvements in BDNF production in both SH-SY5Y cells and mice treated with LPS to stimulate neuroinflammation. Interestingly, in the mice, the levels of IL-1β, IL-6, and TNF-α were reduced in a statistically significant manner upon treatment with Olea.

Carpi et al. [11] showed how Olea and Oleo were able to modulate the expression of *IL-1β*, *COX-2*, *NOX-4*, and *NOX-2* in LPS-stimulated adipocytes. In addition, both in vitro and in silico, Olea and Oleo appeared to be able to reduce the binding between the transcription factor p65 and the target sequence on the *NFKB* gene in pro-inflammatory conditions. Montoya et al. [31] described the regulation of Oleo on pro-inflammatory cytokines and ROS and nitrite production. Further references on the anti-inflammatory effect of Oleo are summarized in [10]. Finally, oils rich in Oleo and Olea have been shown to modulate the inflammatory and oxidative status of patients with obesity in clinical trials [32].

## 4. Materials and Methods

### 4.1. Materials Used

Oleo and Olea were purchased from Active-Italia S.r.l. (Rome, Italy). A 1:1 mixture of DMEM/F12 (without phenol red) was purchased from Microgem S.R.L. (Naples, Italy). L-glutamine at 200 mM and penicillin–streptomycin solution (10,000 U/mL) were purchased from Biosigma (Cona, Italy), while fetal bovine serum was purchased from Gibco (ThermoFisher, Milano, Italy). A Promega^®^ GoTaq^®^ 2-Step RT-qPCR kit was obtained from Promega Corporation (Milan, Italy). All other reagents were purchased from Sigma-Aldrich (Milan, Italy).

### 4.2. Adduct Formation and Chromatographic Analysis

Stock solutions of 50 mM Olea or Oleo were prepared in DMSO. Compounds were diluted in water at a final concentration of 1 mM and incubated with 0.2 µm filtered Tris HCl (pH 7.4) at molar ratios of 1:2, 1:5, 1:10, and 1:100. The adduct formation was found to be immediate, and for this reason, the sample was analyzed immediately after mixing the reagents. After starting the adduct formation, the progress of the reaction was monitored by analyzing the same sample again after 17 min, as this was the time required to complete the chromatographic run.

For the long-term experiments (24 h), the Olea- or Oleo-Tris solutions were maintained statically in thermostatically controlled chambers at 4 °C, 25 °C, or 37 °C. The thermo-stability experiments were repeated at least two times (n = 2).

To assess the stability of the polyphenols in DMEM/F12 and HBSS, Olea, Oleo, or their respective Tris adducts were solubilized in medium and injected onto the UPLC system immediately after preparation or after 180 min. HBSS was prepared as described in [33]. The experiments were repeated at least two times (n = 2).

Chromatographic analyses were performed using a Waters Acquity UPLC system equipped with a quaternary solvent manager (QSM), a PDA detector set at a wavelength of 280 nm, and an ESI–quadrupole. The analyses were performed using a Kinetex C18 EVO column with the following conditions: 2.6 µm, 100 Å, 2.1 × 100 mm (Phenomenex, Torrance, CA, USA), thermostatically controlled at 35 °C, and using the following gradient: A = water + 0.1% formic acid and B = methanol + 0.1% formic acid (Table 2).

The flow rate was set to 0.5 mL/min, and 3 µL of sample was injected. The peak identity was confirmed by the mass spectrometer analyses in the total ion current (TIC) and selected ion recording (SIR) modes (Olea and Oleo in the negative mode, Olea-Tris and Oleo-Tris in the positive mode).

### 4.3. High-Resolution MS and NMR Analysis

High-resolution mass spectra were obtained using a Q Exactive UHMR Hybrid Quadrupole-Orbitrap mass spectrometer (Thermo Fisher, Milano, Italy) with a nano-ESI source (positive mode). These samples were directly introduced into the mass spectrometer using gold-coated capillary needles prepared in-house [34]. The instrument parameters used for MS spectra collection were as follows: capillary voltage of 1.6 kV, scan range from 350 to 1000 *m*/*z*, HCD collision voltage, source fragmentation, and in-source trapping of 0 V. The ion transfer optics were set as follows: injection flatapole of 5 V, inner-flatapole lens of 4 V, bent flatapole of 2 V, and transfer multipole of 0 V. The resolution of the instrument was 35,000 at *m*/*z* = 200, the nitrogen pressure in the HCD cell was maintained at approximately 9.8 × 10^–11^ mbar, and the source temperature was kept at 100 °C. Calibration of the instrument was performed using a 2 mg/mL solution of cesium iodide in water. Solutions of Olea or Oleo with Tris HCl were prepared in DMSO at a 1/2 (secoiridoid/Tris HCl) molar ratio. After 1 h of stirring, the solutions were analyzed with MS.

The ^1^H-NMR experiments were carried out at 298 K on a Bruker Avance (^1^H: 400.13 MHz) Spectrometer (Bruker, Billerica, MA, USA). Each pulse program (^1^H-NMR, selective gradient TOCSY, ^1^H-^1^H COSY, ^1^H-^13^C HSQC) was taken from the available TopSpin 3.5.2 software library. Splitting patterns were designated as s (singlet), d (doublet), t (triplet), q (quartet), or m (multiplet). The ^1^H-NMR spectra of Olea/Oleo (at 2 mg/mL) and Tris HCl (5 mg/mL, pH 5.8) were recorded in DMSO-d_6_ and D_2_O respectively. Then, a mixture of Olea/Oleo and Tris HCl (pH 5.8), with a molar ratio of 1/2, in D_2_O was prepared in the NMR tube, and the reaction mixture was monitored over time.

The adduct of Oleo-Tris was isolated by multiple injections into the UHPLC system with the same elution conditions as those reported in the LC-MS section without using the acid additive in order to preserve the integrity of the adduct. The collected fractions were quickly lyophilized and dissolved in D_2_O for NMR analysis.

### 4.4. DPPH Assay

A 120 µM solution of 2,2-diphenyl-1-picrylhydrazyl (DPPH·) was prepared by dissolving an appropriate amount of powder in ethanol and diluiting until a spectrophotometric absorbance value of 0.7 at 517 nm was reached. To 1 mL of DPPH· solution, 20 µL of each sample at various concentrations (0–1 mM) was added. The reaction was allowed to proceed in the dark at room temperature for 15 min, and the absorbance values were recorded at 517 nm using ethanol as a blank. The calculated IC_50_ values, expressed in µM, were compared with the values obtained from the ascorbic acid calibration curve. The experiments were conducted at least two times (n = 2).

### 4.5. ABTS Assay

The 2,2′-azinobis(3-ethylbenzothiazoline-6-sulfonic acid) diammonium salt (ABTS) was dissolved in water to reach a final concentration of 7 mM. The ABTS radical cation (ABTS·⁺) was generated by adding 2.45 mM potassium persulfate. The mixture was incubated in the dark for 16 h and subsequently diluted with ethanol to achieve an absorbance value of 0.7 at 734 nm. To 1 mL of ABTS·⁺ solution, 20 µL of each sample at various concentrations (0–1 mM) was added. The reaction was allowed to proceed in the dark at room temperature for 6 min, and the absorbance values were recorded at 734 nm using ethanol as a blank. The calculated IC_50_ values, expressed in µM, were compared with the values obtained from the ascorbic acid calibration curve. The experiments were conducted at least two times (n = 2).

### 4.6. Biological Experiment on BV-2 Cell Line

The murine microglial cell line BV-2 was cultured in Dulbecco’s modified eagles medium / F12 (1:1) (DMEM/F12) medium supplemented with 10% fetal bovine serum (FBS), 4 mM L-glutamine, and 1% penicillin–streptomycin solution and kept in an incubator maintained at 37 °C, 5% CO_2_, and 99% RH. All the experiments were performed between the 5–15th cell passage.

For the experiment in HBSS, BV-2 cells were seeded in 6-well plates at a density of 600,000 cells per well. After 24 h, the medium was replaced by HBSS, and the cells were treated with 1 µg/mL of LPS and 10 µM of Olea, Oleo, or their respective Tris adducts prepared 24 h before and maintained at 4 °C until treatment. The same amount of Tris present in the adduct was administered as a control in the LPS treatment. Non-stimulated cells were treated with DMSO as a control for the polyphenols and with water as control for the LPS. After 6 h of incubation, the cells were lysed with TRI-REAGENT and stored at −80 °C until analysis.

For the experiment in DMEM/F12, BV-2 cells were seeded in 6-well plates at a density of 90,000 cells per well. After 24 h, the cells were treated with 1 µg/mL of LPS and 10 µM of Olea/Oleo or their adducts prepared 24 h before and maintained at 4 °C until the treatment. The same amount of Tris present in the adduct was administered as a control in the LPS treatment. Non-stimulated cells were treated with DMSO as a control for the polyphenols and with water as control for the LPS. After 24 h of incubation, the cells were lysed with TRI-REAGENT and stored at −80 °C until analysis.

RNA extraction was performed according to the manufacturer’s protocol, and the mRNA was quantified and retro-transcribed in cDNA using a Promega^®^ GoTaq^®^ 2-Step RT-qPCR kit (Promega Corporation, Madison, WI, USA). qPCR was then performed on a CFX device (BIORAD^®^, Hercules, CA, USA) using the SYBR Green fluorescent stain (Promega^®^, Madison, WI, USA). The expression of murine *IL-1β* and *iNOS* transcripts was normalized on murine Ribosomal Protein S27A (*RPS27A*) transcript, and the ΔΔCt method was used to calculate the relative gene expression. The melting curves were used in order to verify the presence of a single PCR product. The primers used in the qPCR reactions can be found in Table 3. Biological experiments were conducted at least three times (n = 3).

### 4.7. Statistical Analysis

The software programs EXCEL^®^ and GraphPad Prism 8.0 were used to analyze and plot the data. All the results are presented as mean ± standard deviation (SD) or ±standard error of mean (SEM). The analysis of statistical significance was performed using the unpaired *t*-test. The level of significance was set at *p* < 0.05.

## 5. Conclusions

Taken together, our data confirmed the evidence described by Darakjian and col-leagues regarding the ability of Oleo to form adducts with molecules containing primary amines. This evidence was also extended to Olea. Furthermore, this ability appeared to extend not only to biogenic amines such as amino acids but also to non-biogenic amines such as Tris. The degradation rate of the adducts, and therefore their intrinsic instability, makes it difficult to envision a pharmacological application for these new molecules. However, their study has shed light on the mechanisms of action of Olea and Oleo in a biological system. Once administered, these polyphenols can react unpredictably with a wide range of molecules containing primary amine groups, yet their antioxidant and anti-inflammatory effects remain active. Our data suggest that, at the tested concentrations and for the investigated genes, these effects are not mediated by their degradation products, Ty and HTy, but rather by the adducts themselves. All of this, in combination with the findings of Darakjian and colleagues, opens a new interpretative framework for understanding the numerous pieces of evidence regarding the properties of Olea and Oleo in vivo.

## Figures and Tables

**Table 1:** DPPH· and ABTS·^+^ antioxidant activity assays, expressed as IC_50_ (µM) and compared with ascorbic acid.

	Ascorbic Acid	Olea	Oleo	Olea-Tris	Oleo-Tris	Olea 24 h	Oleo 24 h	Olea-Tris 24 h	Oleo-Tris 24 h
DPPH mean ± SD	34.00 ± 0.56	60.00 ± 4.70	5456 ± 1296	60.00 ± 2.80	331.00 ± 51.00	86.00 ± 32.00	5541 ± 1503	265.00 ± 77.00	6303.00 ± 151.00
ABTS·^+^ mean ± SD	6.00 ± 0.95	43.00 ± 2.60	92.00 ± 16.00	37.00 ± 2.30	4.60 ± 0.46	45.00 ± 8.70	105.00 ± 3.00	59.00 ± 16.00	5.60 ± 0.28

**Table 2:** Chromatographic gradient scheme.

Time	% Water + 0.1% Formic Acid	% Methanol + 0.1% Formic Acid
0 min	98	2
1 min	98	2
6 min	45	55
10 min	20	80
10.5 min	0	100
12.5 min	0	100
12.7 min	98	2
17 min	98	2

**Table 3:** Primers used in qPCR analysis.

Target	Forward	Tm (°C)	Reverse	Tm (°C)
*RPS27A*	AGA GGC TGA TCT TTG CTG GT	59	ACC AGA TGA AGG GTG GAC TC	59
*IL-1β*	TTC GTG AAT GAG CAG ACA GC	59	CCA TGG TTT CTT GTG ACC CT	58
*iNOS*	GCA AGA GAG TGC TGT TCC AG	60	CCT GAA CGT AGA CCT TGG GT	59

## Data Availability

The original contributions presented in this study are included in the article/Supplementary Materials. Further inquiries can be directed to the corresponding author(s).

## Supplementary Materials

The following supporting information can be downloaded at: https://www.mdpi.com/article/10.3390/molecules30071645/s1. Figure S1: HRMS zoomed spectra for signal 426 *m*/*z* of Oleo + Tris HCl mixture. Figure S2: HRMS zoomed spectra for signal 426 *m*/*z* of Olea + Tris HCl mixture. Figure S3: ^1^H NMR-spectrum (400 MHz, D_2_O) of Tris HCl (pH: 5.8) without water suppression signal. Figure S4: ^1^H NMR-spectrum (400 MHz, D_2_O) of Tris HCl (pH: 5.8) water suppression (ws) signal. Figure S5: ^1^H NMR-spectrum (400 MHz, DMSO-*d_6_*) of Tris HCl (pH = 5.8): zoomed view. Figure S6: ^1^H NMR-spectrum (400 MHz, DMSO-*d_6_*) of Oleacin. Figure S7: 1D Selective Gradient TOCSY freq: 1.981ppm NMR-spectrum (400 MHz, DMSO-*d_6_*) of Oleacin. Figure S8: ^1^H NMR-Spectrum (400 MHz, DMSO-*d6*/D_2_O) of Oleocanthal. Figure S9: 1D Selective Gradient TOCSY freq: 1.907ppm NMR-spectrum (400 MHz, D_2_O) of Oleocanthal. Figure S10: ^1^H NMR-Spectrum (400 MHz, D_2_O) of Oleacin (ws). Figure S11: ^1^H NMR-spectrum (400 MHz, D_2_O) of Oleacin and Tris HCl after 10 min (ws). Figure S12: ^1^H NMR-spectrum (400 MHz, D_2_O) of Oleacin and Tris HCl after 35 min (ws). Figure S13: ^1^H NMR-spectrum (400 MHz, D_2_O) of Oleacin and Tris HCl after 60 min (ws). Figure S14: ^1^H NMR-spectrum (400 MHz, D_2_O) of Oleacin and Tris HCl 270 min (ws). Figure S15: ^1^H NMR-spectrum (400 MHz, D_2_O) of Oleacin and Tris HCl after 24 h (ws). Figure S16: Overview of ^1^H-NMR of Olea/Tris HCl mixture in D_2_O over time. Figure S17: 2D-COSY of Oleacin and Tris HCl mixture in DMSO-*d6*/D_2_O after 350 min. Figure S18: Zoom of 2D COSY of oleacin and Tris HCl mixture in DMSO-*d6*/D_2_O after 350 min. Figure S19: ^1^H NMR-Spectrum (400 MHz, D_2_O) of Oleocanthal (ws). Figure S20: ^1^H NMR-spectrum (400 MHz, D_2_O) of Oleocanthal and Tris HCl after 30 min (ws). Figure S21: ^1^H NMR-spectrum (400 MHz, D_2_O) of Oleocanthal and Tris HCl after 60 min (ws). Figure S22: ^1^H NMR-spectrum (400 MHz, D_2_O) of Oleocanthal and Tris HCl after 180 min (ws). Figure S23: ^1^H NMR-spectrum (400 MHz, D_2_O) of Oleocanthal and Tris HCl after 300 min (ws). Figure S24: ^1^H NMR-spectrum (400 MHz, D_2_O) of Oleocanthal and Tris HCl after 24 h (ws). Figure S25: HSQC of Oleocanthal and Tris HCl mixture in D_2_O after 300 min. Figure S26: Zoom of HSQC of Oleocanthal and Tris HCl mixture in D_2_O. Figure S27: Zoom of HSQC of Oleocanthal and Tris HCl mixture in D_2_O. Figure S28: 1D Selective Gradient TOCSY freq: 2.015ppm (400 MHz, D_2_O) of Oleocanthal and Tris HCl after 300 min. Figure S29: ^1^H NMR-spectrum (400 MHz, D_2_O) of Oleocanthal adduct. Figure S30: ^1^H NMR-spectrum (400 MHz, D_2_O) of Oleocanthal adduct signal zoomed view. Figure S31: ^1^H NMR-spectrum (400 MHz, D_2_O) of Oleocanthal adduct with water suppression signal. Figure S32: 1D Selective Gradient TOCSY freq: 1.158pp NMR-spectrum (400 MHz, D_2_O) of Oleocanthal adduct. Figure S33: 1D Selective Gradient TOCSY freq: 1.544ppm NMR-spectrum (400 MHz, D_2_O) of Oleocanthal adduct. Figure S34: 2D COSY of Oleocanthal adduct and Tris HCl mixture in D_2_O. Figure S35: 2D COSY of Oleocanthal adduct and Tris HCl mixture in D_2_O: zoomed view. Figure S36: 2D COSY of Oleocanthal adduct and Tris HCl mixture in D_2_O: zoomed view. Figure S37: ^1^H-NMR of Oleo, Oleo adducts and Oleo + Tris HCl mixture in D_2_O. Figure S38: Mass spectra of Oleo-Tris, extracted from Total-Ion Current (TIC) trace, after the purification process.
