# Supplementary material for: Spontaneous Reaction of Oleacein and Oleocanthal with Primary Amines: A Biochemical Perspective
Source: Molecules. 2025 Apr 7;30(7):1645. doi: 10.3390/molecules30071645 (PMC11990156; doi:10.3390/molecules30071645)

Supplementary Info

Spontaneous Reaction of Oleacein and Oleocanthal with Primary Amines: A Biochemical Perspective

Daniel Di Risola [1,†], Davide Laurenti [1,†], Francesca Ferraro [1], Alessia Ciogli [2], Simone Manetto [2], Yuri Gazzilli [2], Rodolfo Federico [3], Antonio Francioso [4], Luciana Mosca [1,*] and Roberto Mattioli [1,*]

[1] Department of Biochemical Sciences "A. Rossi Fanelli", Sapienza University of Rome, pl.e Aldo Moro 5, 00185 Rome, Italy; daniel.dirisola@uniroma1.it (D.D.R.); davide.laurenti@uniroma1.it (D.L.); francescaferraro998@gmail.com (F.F.)
[2] Department of Chemistry and Technology of Drugs, Sapienza University of Rome, pl.e Aldo Moro 5, 00185 Rome, Italy; alessia.ciogli@uniroma1.it (A.C.); simone.manetto@uniroma1.it (S.M.); yuri.gazzilli@uniroma1.it (Y.G.)
[3] Active-Italia S.r.l., Via delle Terme Deciane 10, 00153 Rome, Italy; federico@active-italia.com
[4] Department of Bioscience and Technology for Food Agriculture and Environment, University of Teramo, 64100 Teramo, Italy; afrancioso@unite.it
* Correspondence: luciana.mosca@uniroma1.it (L.M.); roberto.mattioli@uniroma1.it (R.M.)
† These authors contributed equally to this work.

Contents:

1. **High resolution mass spectra: zoomed view** 2
2. **NMR spectra of starting materials** 3
3. **^{1}H NMR of Olea/Tris HCl mixture in D_2O over time** 7
4. **1H NMR of Oleo/Tris HCl mixture in D_2O over time** 11
5. **NMR spectra of isolated adduct** 18

S1. High resolution mass spectra: zoomed view

Figure S1 HRMS zoomed spectra for signal 426 m/z of Oleo + Tris HCl mixture.

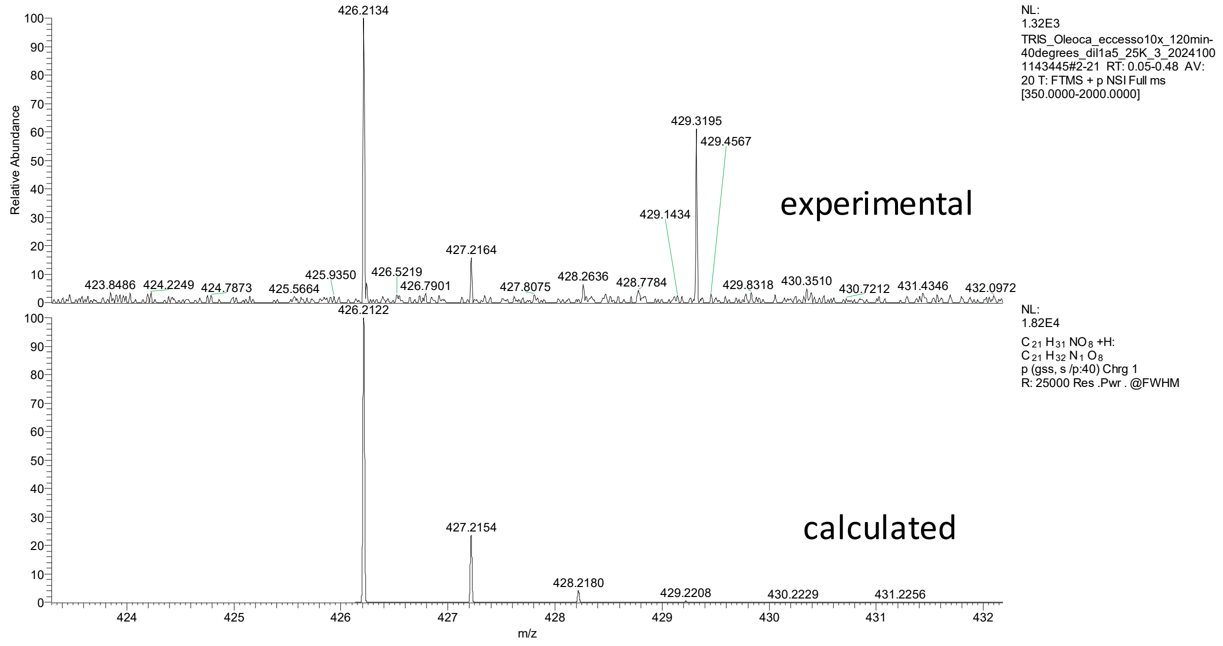

$[M+H]^+$ = 426.2134 m/z, Δm= 2.80 ppm

Figure S2 HRMS zoomed spectra for signal 426 m/z of Olea + Tris HCl mixture.

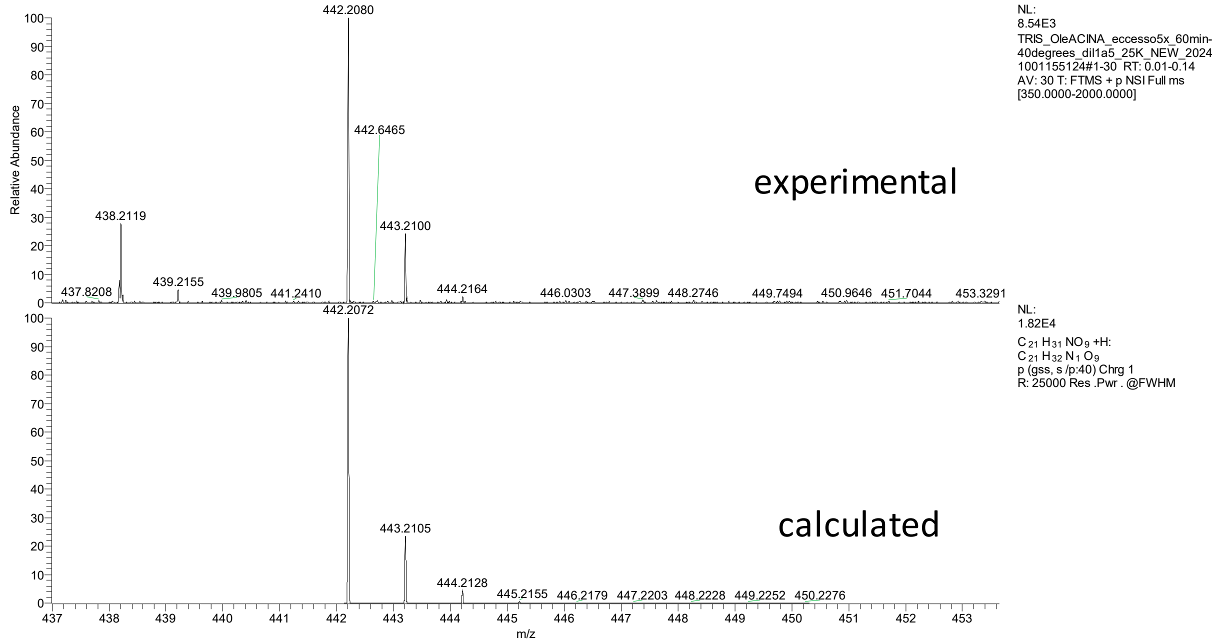

$[M+H]^+$ = 442.2080, Δm= 1.81 ppm

S2. NMR spectra of starting materials

Figure S3 ^{1}H NMR-spectrum (400 MHz, D_2O) of Tris HCl (pH: 5.8) without water suppression signal.

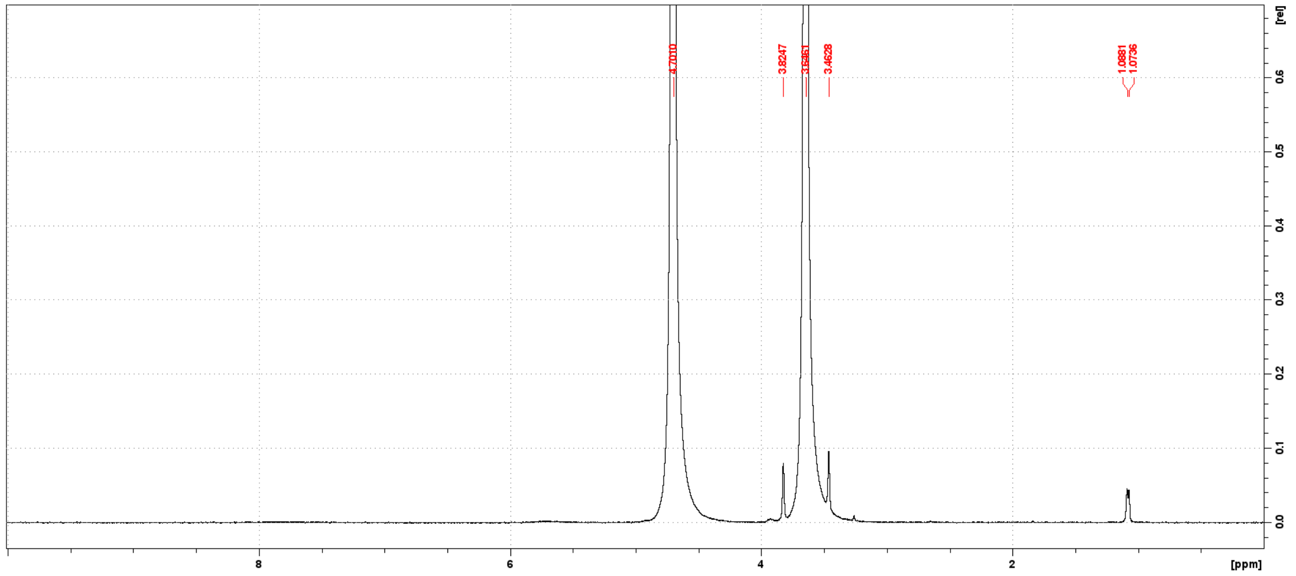

Figure S4 ^{1}H NMR-spectrum (400 MHz, D_2O) of Tris HCl (pH: 5.8) water suppression (ws) signal.

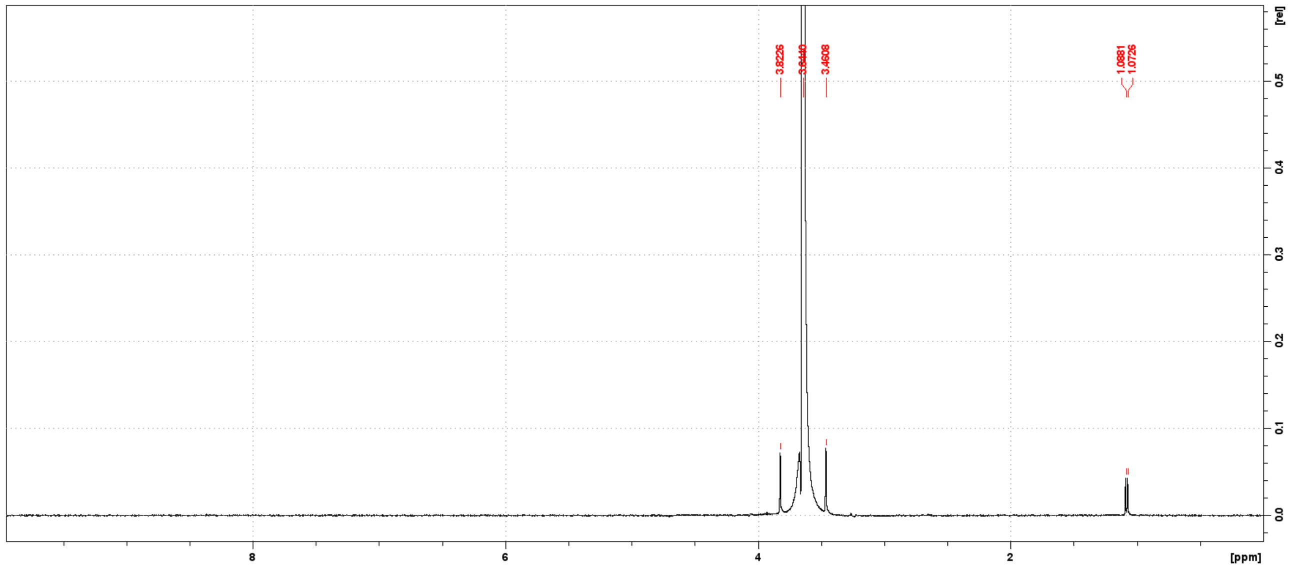

Figure S5 ^{1}H NMR-spectrum (400 MHz, DMSO-d_6) of Tris HCl (pH = 5.8): zoomed view.

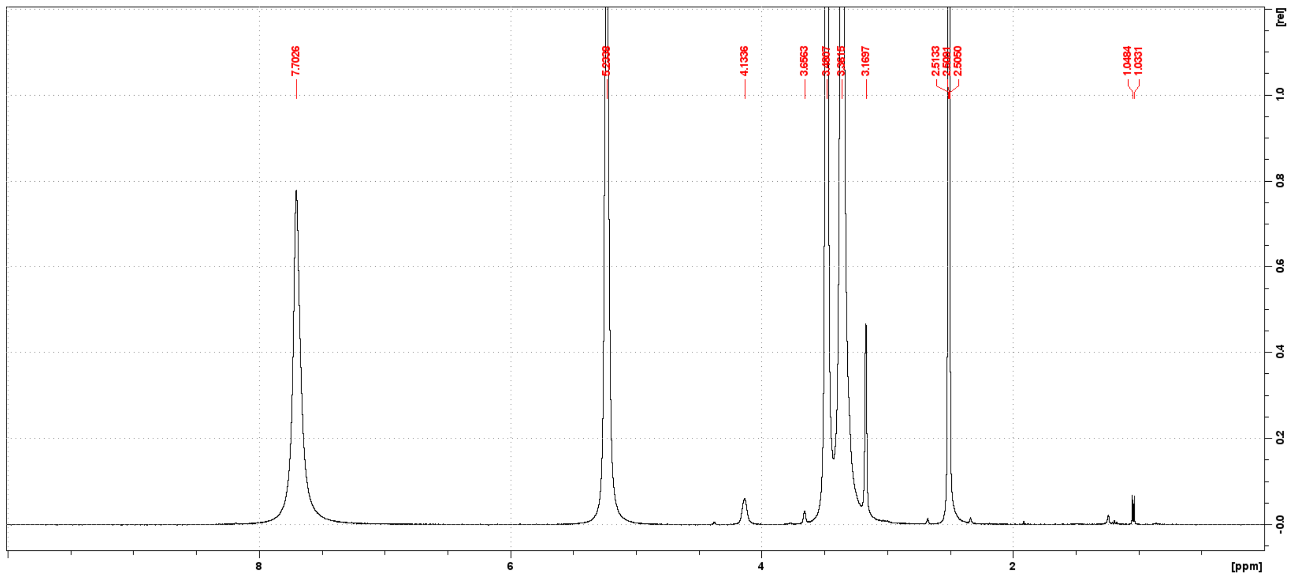

Figure S6 ^{1}H NMR-spectrum (400 MHz, DMSO-d_6) of Oleacin.

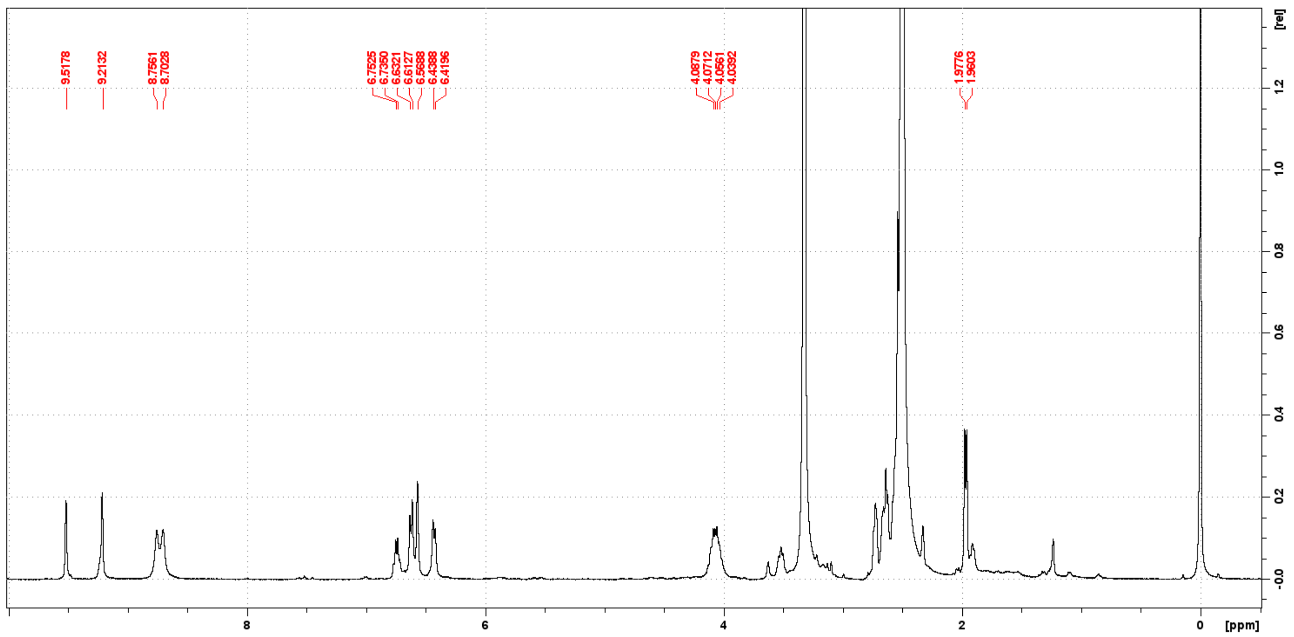

Figure S7 1D Selective Gradient TOCSY freq: 1.981ppm NMR-spectrum (400 MHz, DMSO-d_6) of Oleacin.

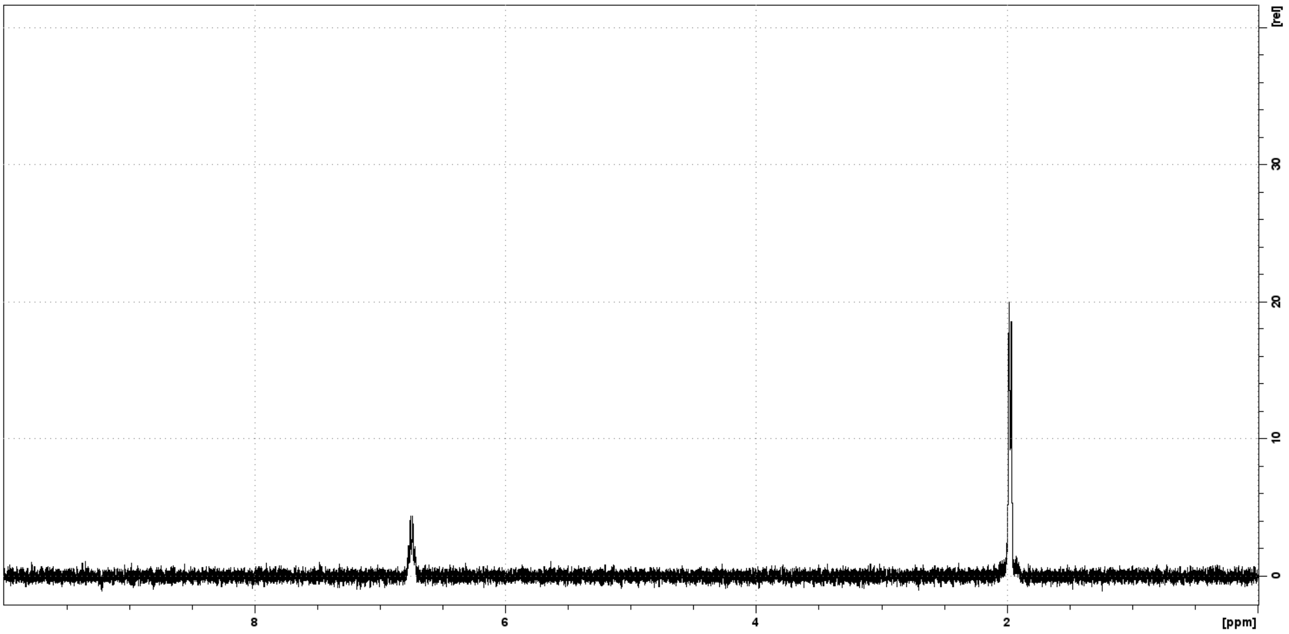

Correlation confirmed between CH_3 at 2.01 ppm (d) and CH at 6.75 ppm (q)

Figure S8 ^{1}H NMR-Spectrum (400 MHz, DMSO-*d6*/D_2O) of Oleocanthal.

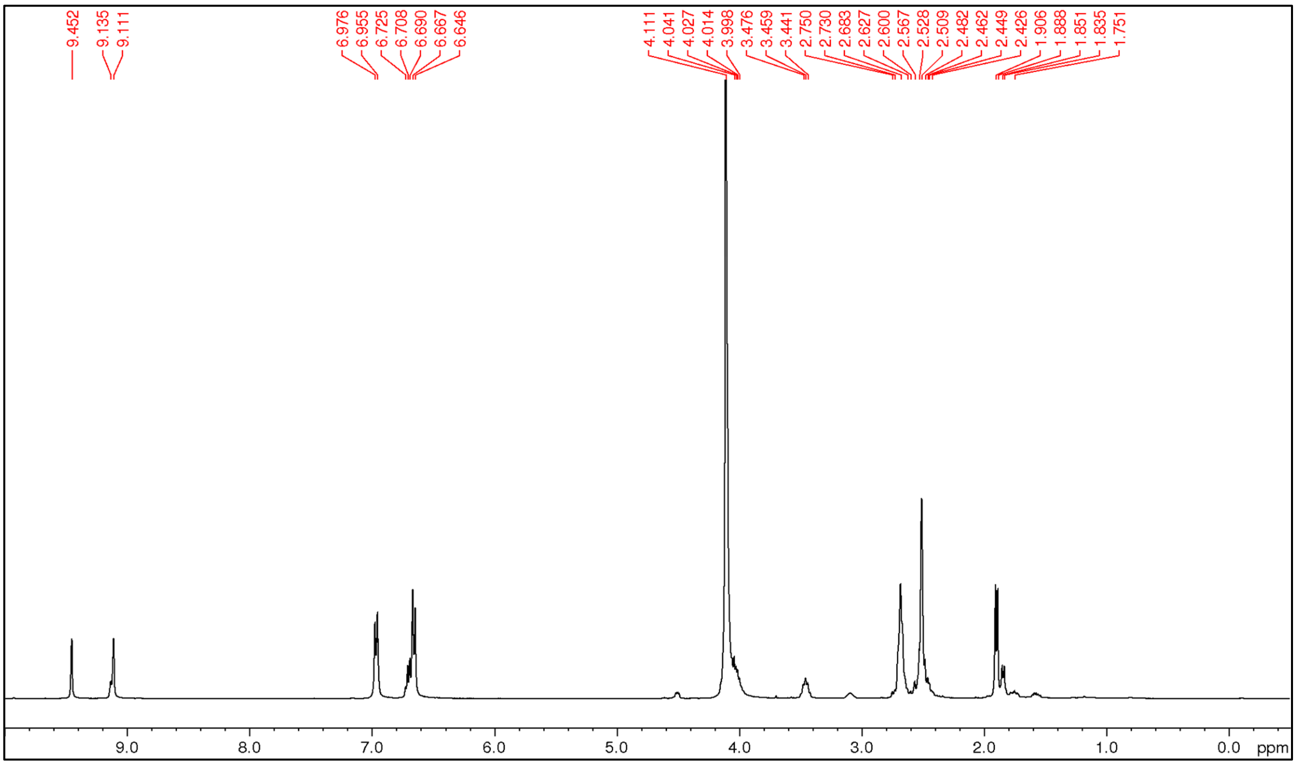

Figure S9 1D Selective Gradient TOCSY freq: 1.907ppm NMR-spectrum (400 MHz, D_2O) of Oleocanthal.

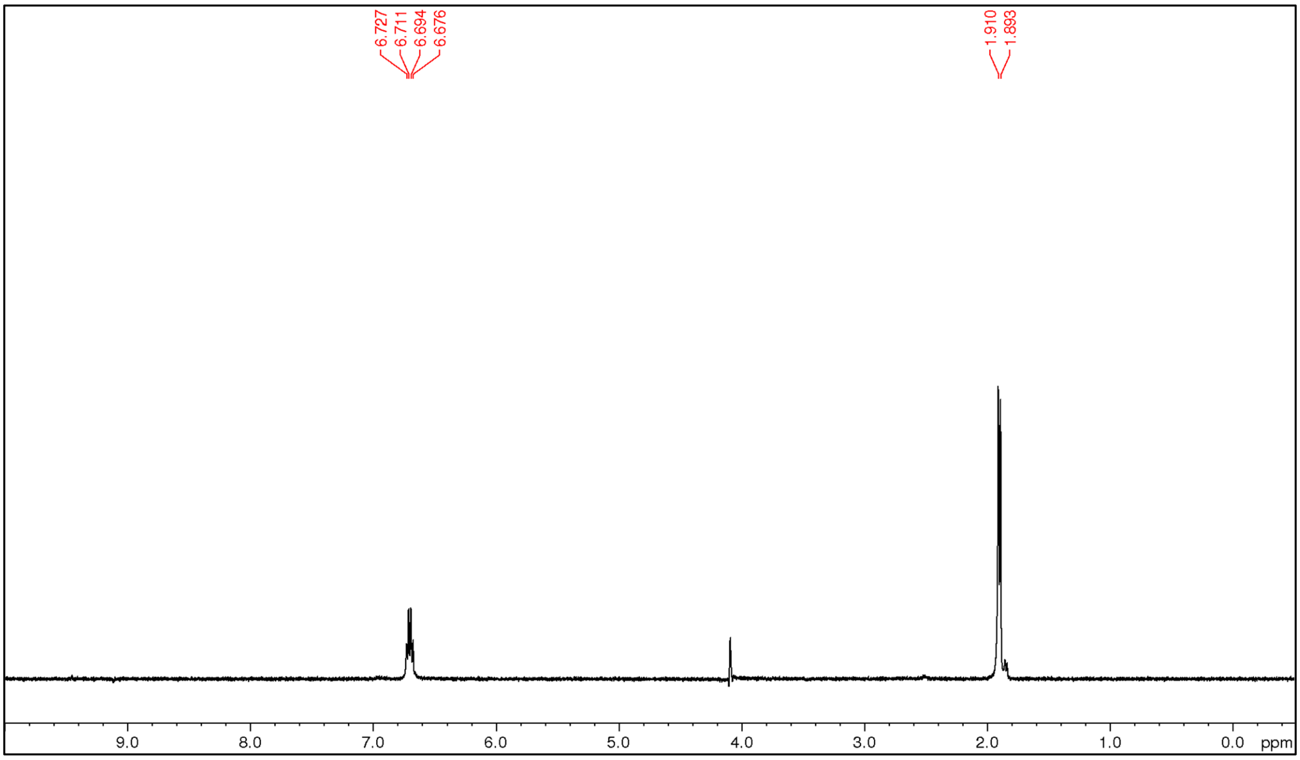

Correlation confirmed between CH_3 at 1.91 ppm (d) and CH at 6.70 ppm (q)

S3. ^{1}H NMR of Olea/Tris HCl mixture in D_2O over time

Figure S10 ^{1}H NMR-Spectrum (400 MHz, D_2O) of Oleacin (ws).

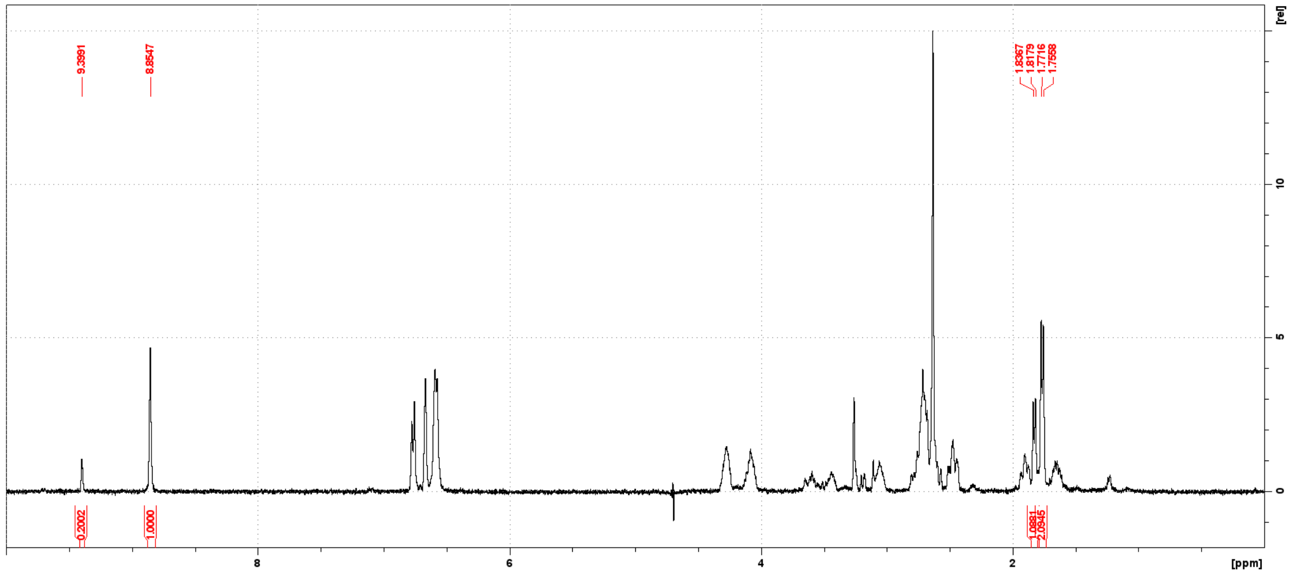

Figure S11 ^{1}H NMR-spectrum (400 MHz, D_2O) of Oleacin and Tris HCl after 10 min (ws).

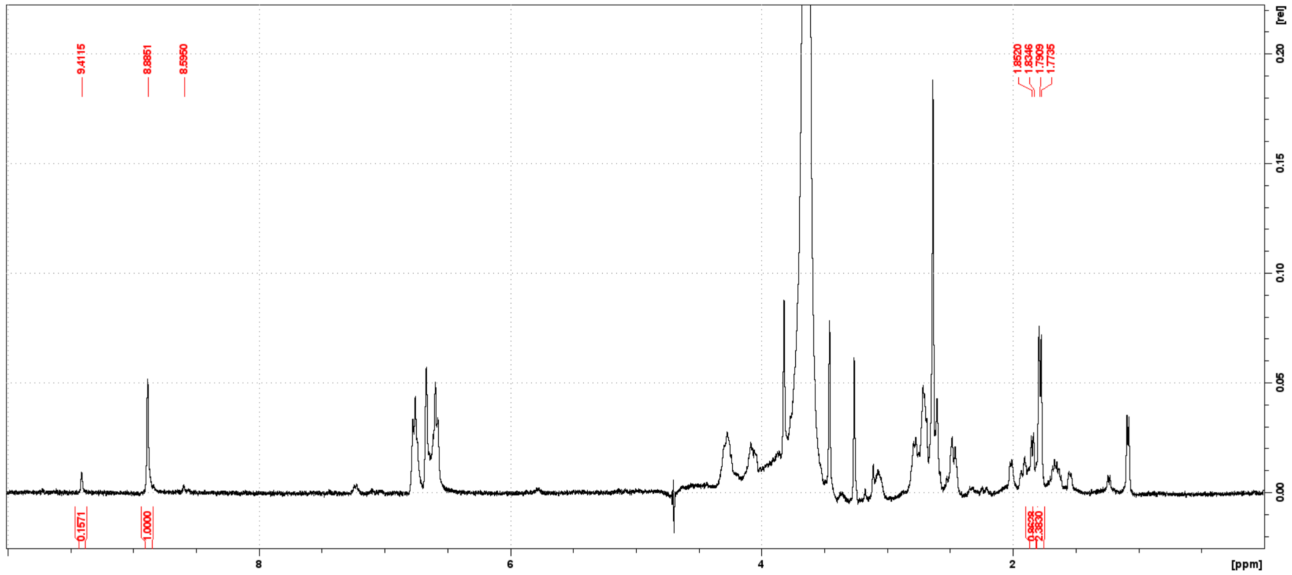

Figure S12 ^{1}H NMR-spectrum (400 MHz, D_2O) of Oleacin and Tris HCl after 35 min (ws).

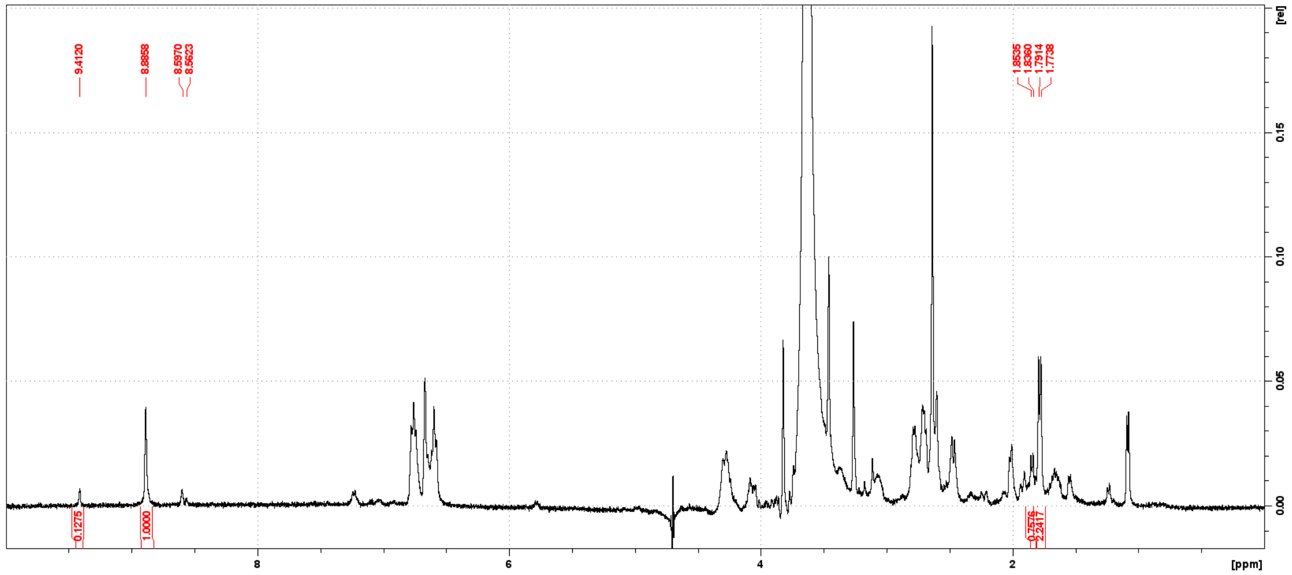

Figure S13 ^{1}H NMR-spectrum (400 MHz, D_2O) of Oleacin and Tris HCl after 60 min (ws).

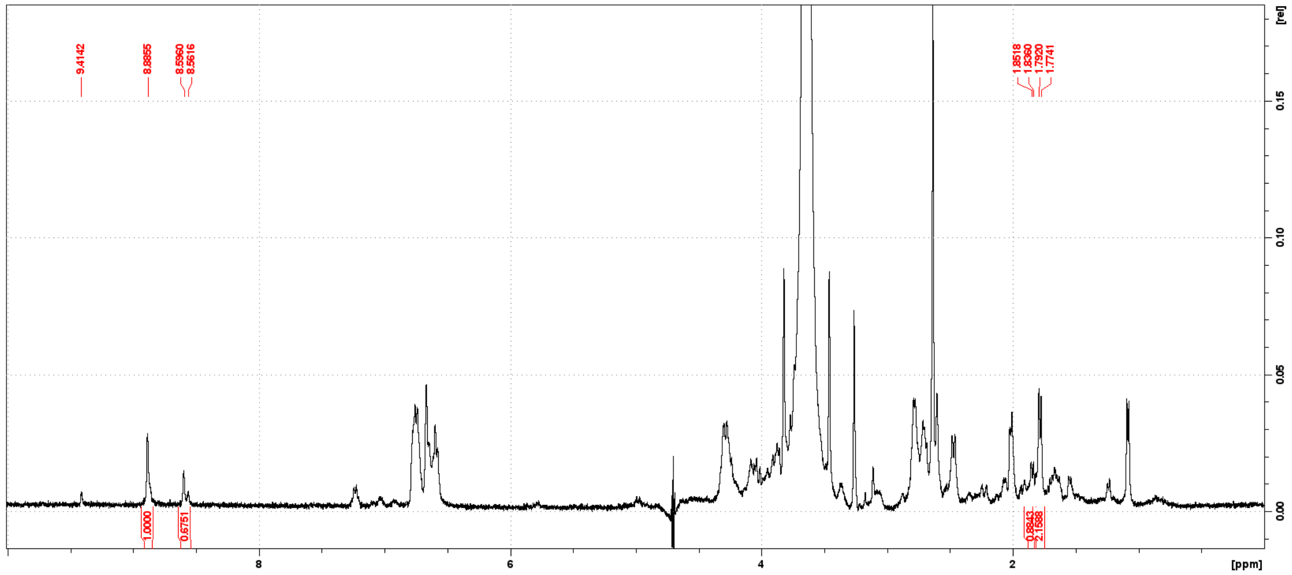

Figure S14 ^{1}H NMR-spectrum (400 MHz, D_2O) of Oleacin and Tris HCl 270 min (ws).

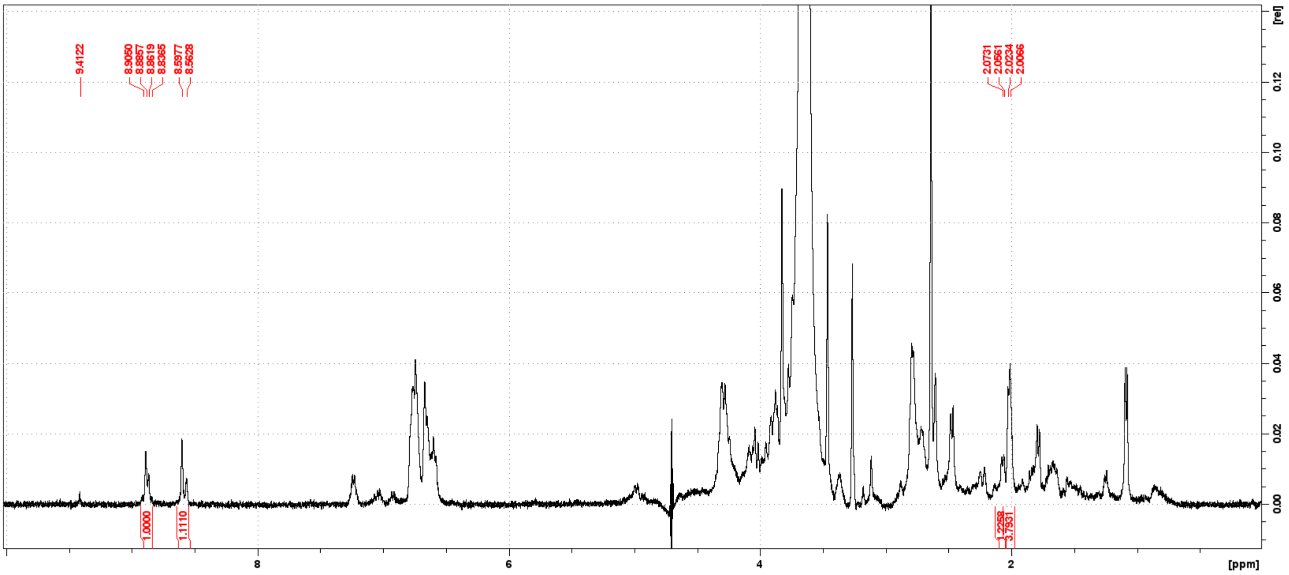

Figure S15 ^{1}H NMR-spectrum (400 MHz, D_2O) of Oleacin and Tris HCl after 24 h (ws).

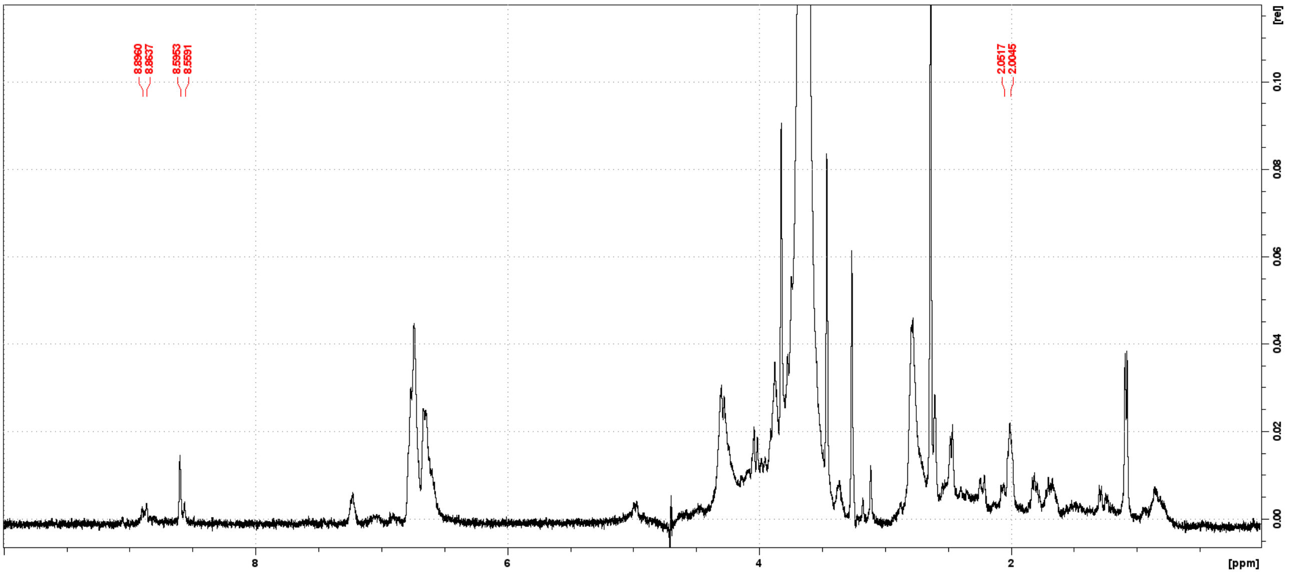

Figure S16. Overview of ^{1}H-NMR of Olea/Tris HCl mixture in D_2O over time. The asterisk highlights the presence of a new signal not related to **A**-**C** structures.

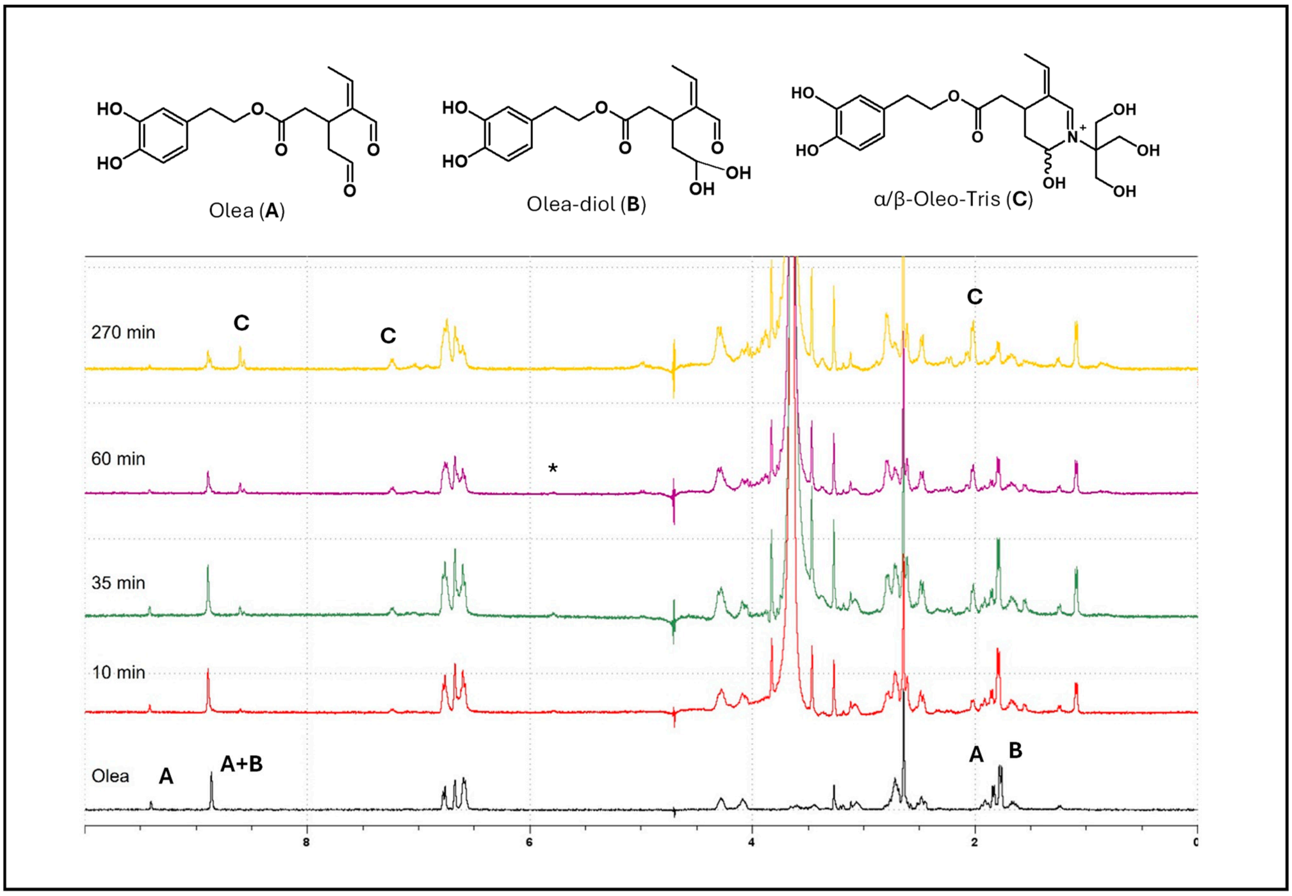

Figure S17 2D-COSY of Oleacin and Tris HCl mixture in DMSO-*d6*/D_2O after 350 min.

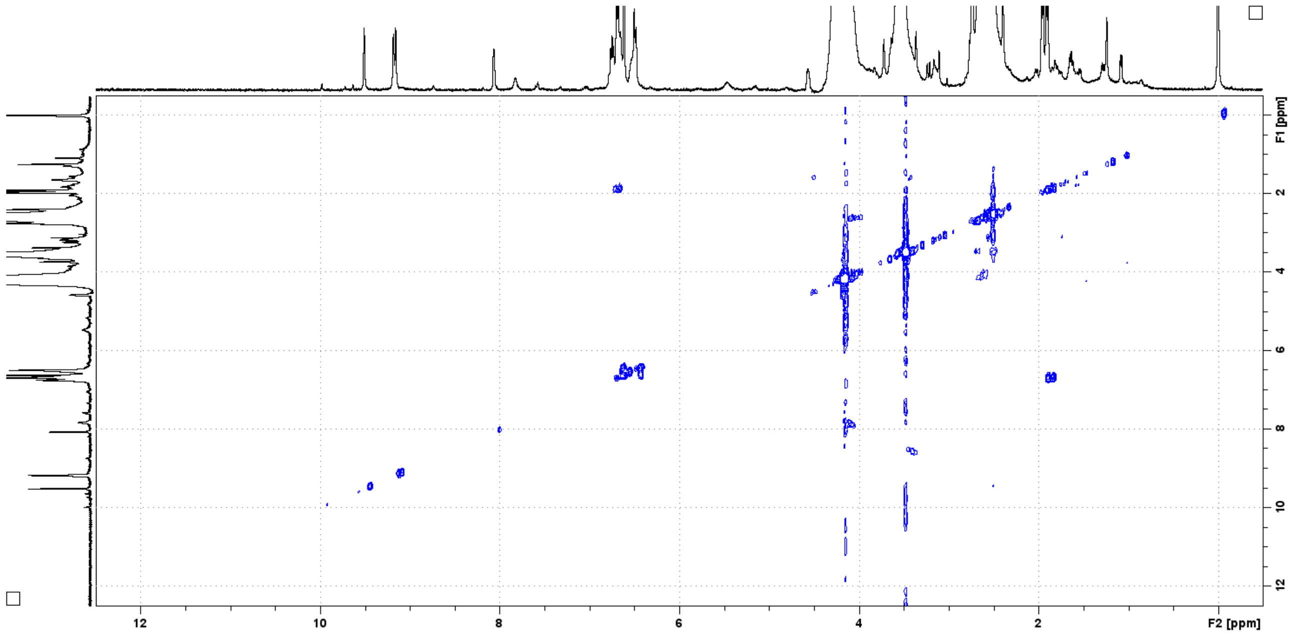

Figure S18 Zoom of 2D COSY of oleacin and Tris HCl mixture in DMSO-*d6*/D_2O after 350 min.

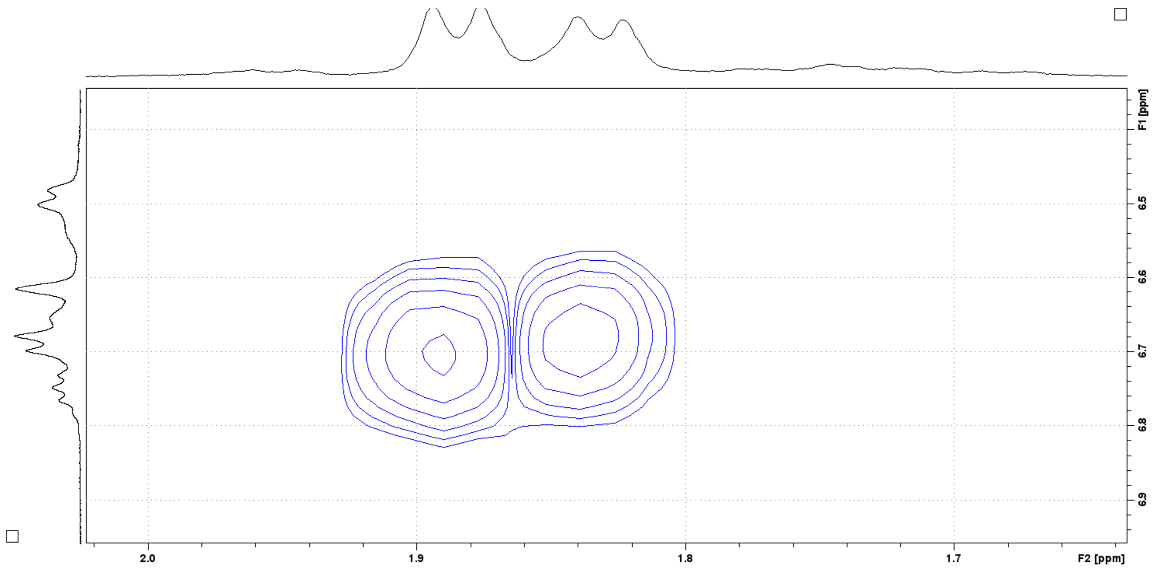

S4. Kinetic of Oleo/Tris HCl mixture in D_2O over time

Figure S19 ^{1}H NMR-Spectrum (400 MHz, D_2O) of Oleocanthal (ws).

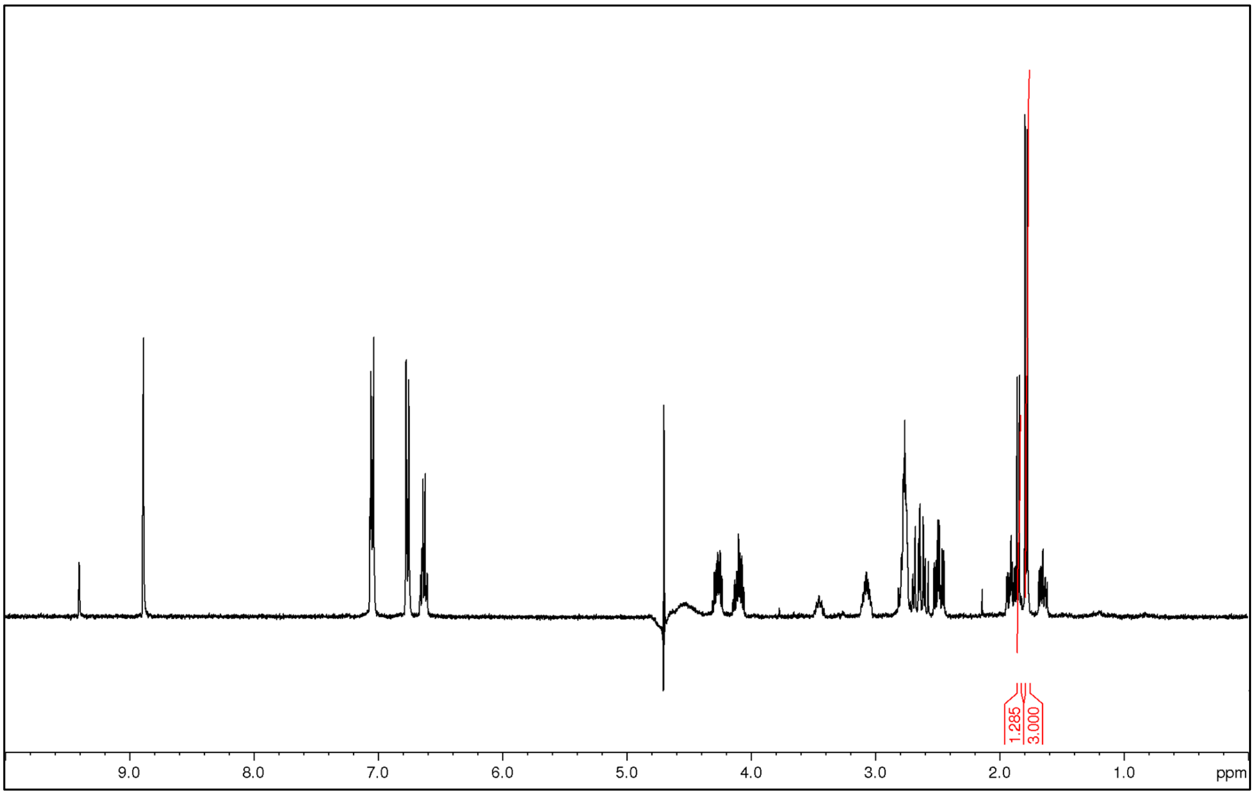

Figure S20 ^{1}H NMR-spectrum (400 MHz, D_2O) of Oleocanthal and Tris HCl after 30 min (ws).

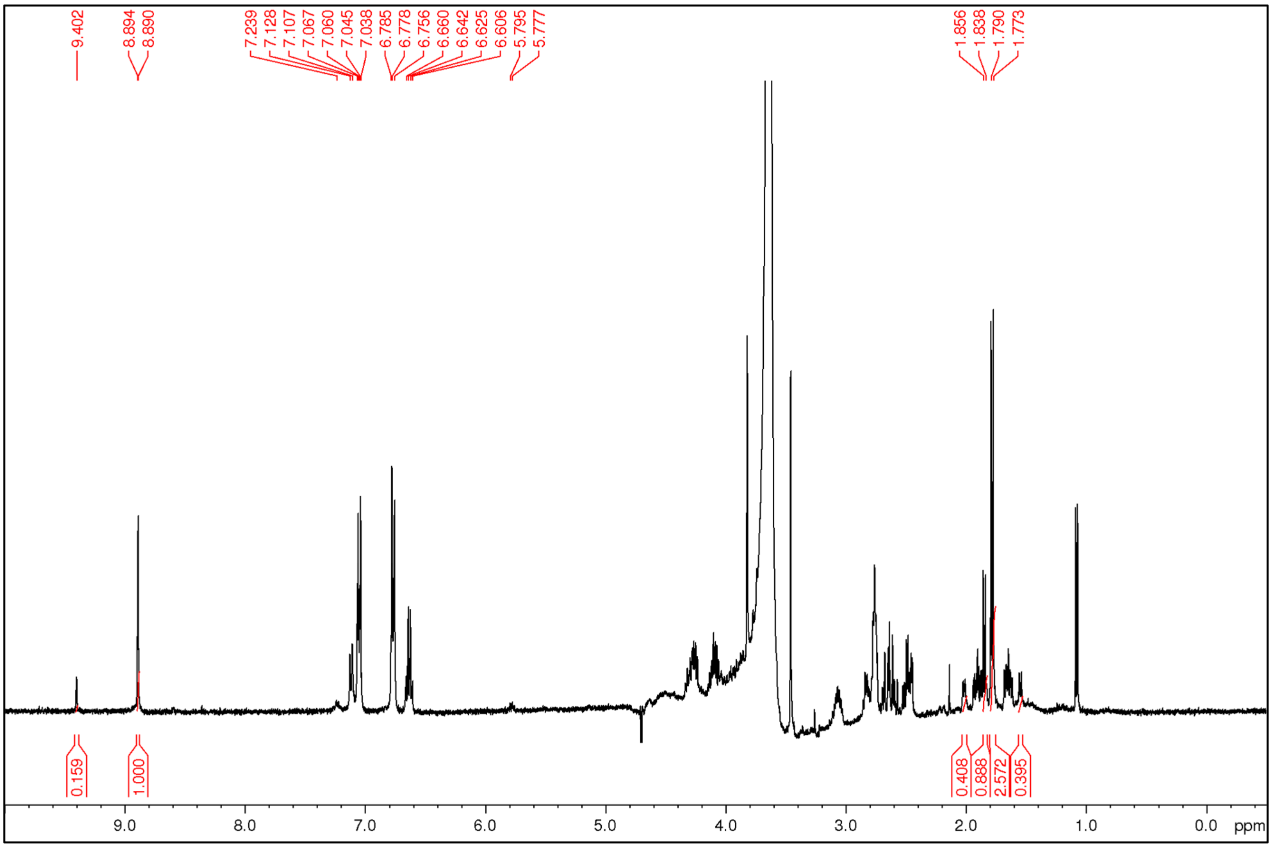

Figure S21 ^{1}H NMR-spectrum (400 MHz, D_2O) of Oleocanthal and Tris HCl after 60 min (ws).

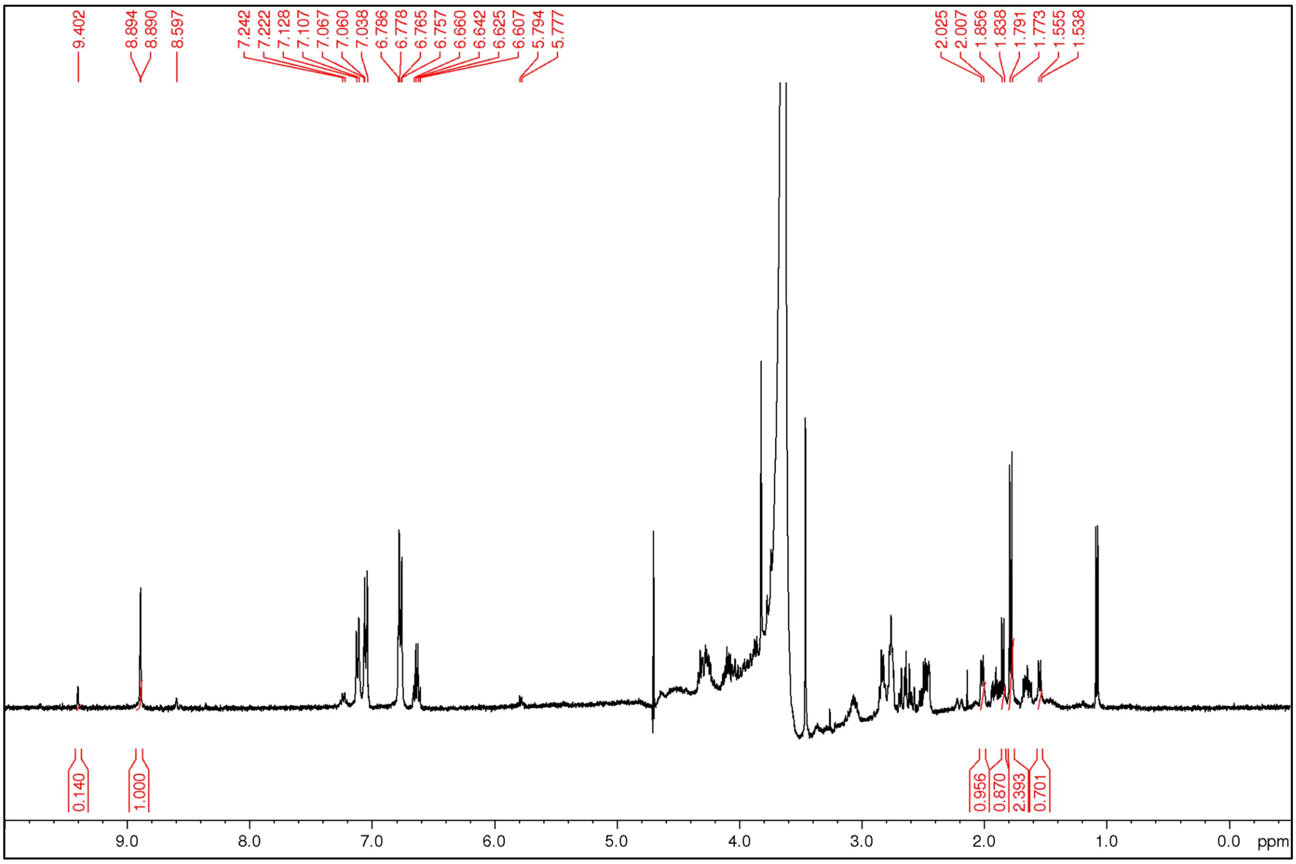

Figure S22 ^{1}H NMR-spectrum (400 MHz, D_2O) of Oleocanthal and Tris HCl after 180 min (ws).

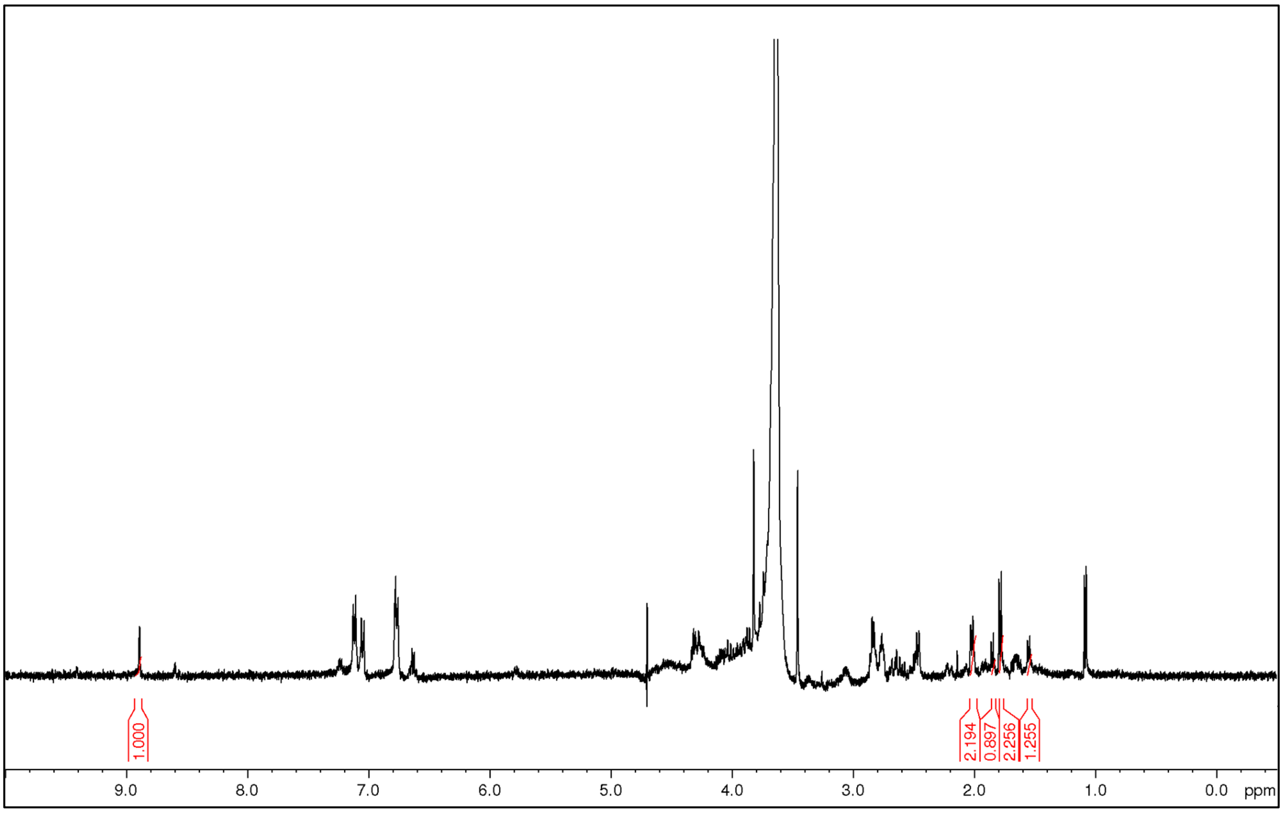

Figure S23 ^{1}H NMR-spectrum (400 MHz, D_2O) of Oleocanthal and Tris HCl after 300 min (ws).

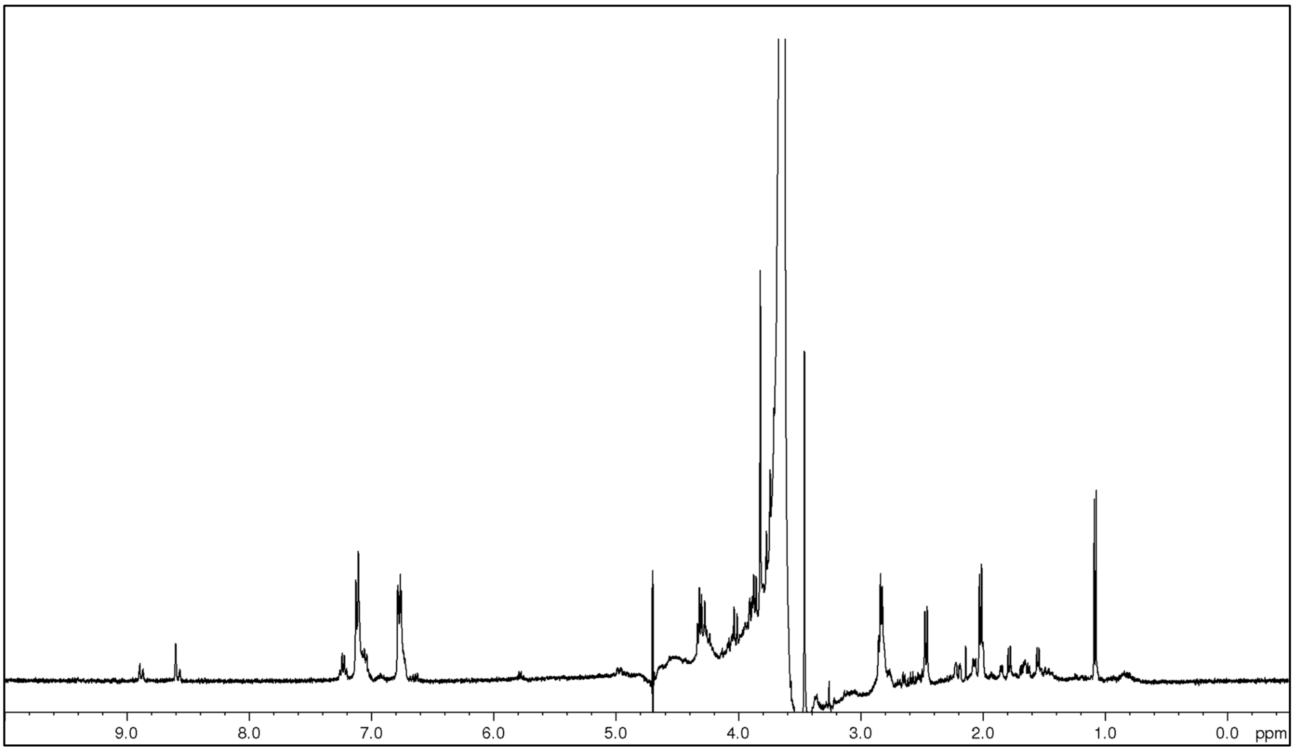

Figure S24 ^1H NMR-spectrum (400 MHz, D_2O) of Oleocanthal and Tris HCl after 24h (ws).

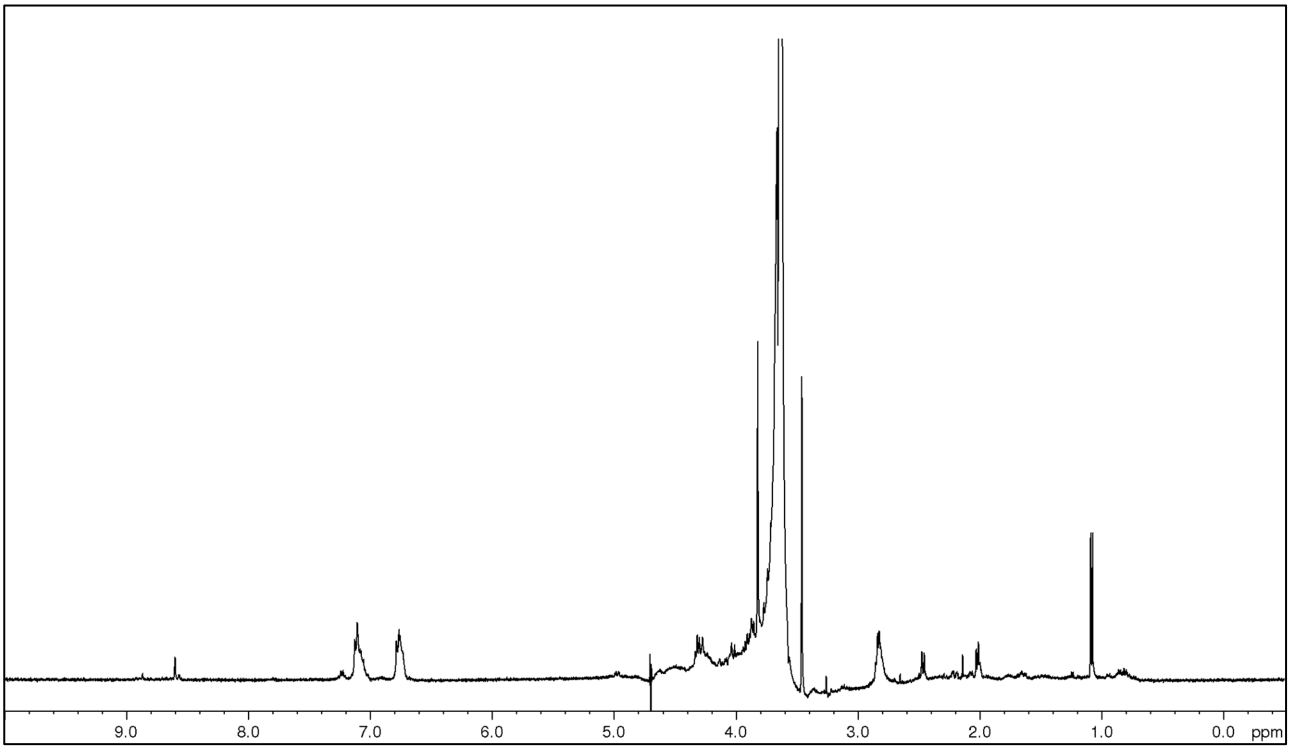

Figure S25 HSQC of Oleocanthal and Tris HCl mixture in D_2O after 300 min.

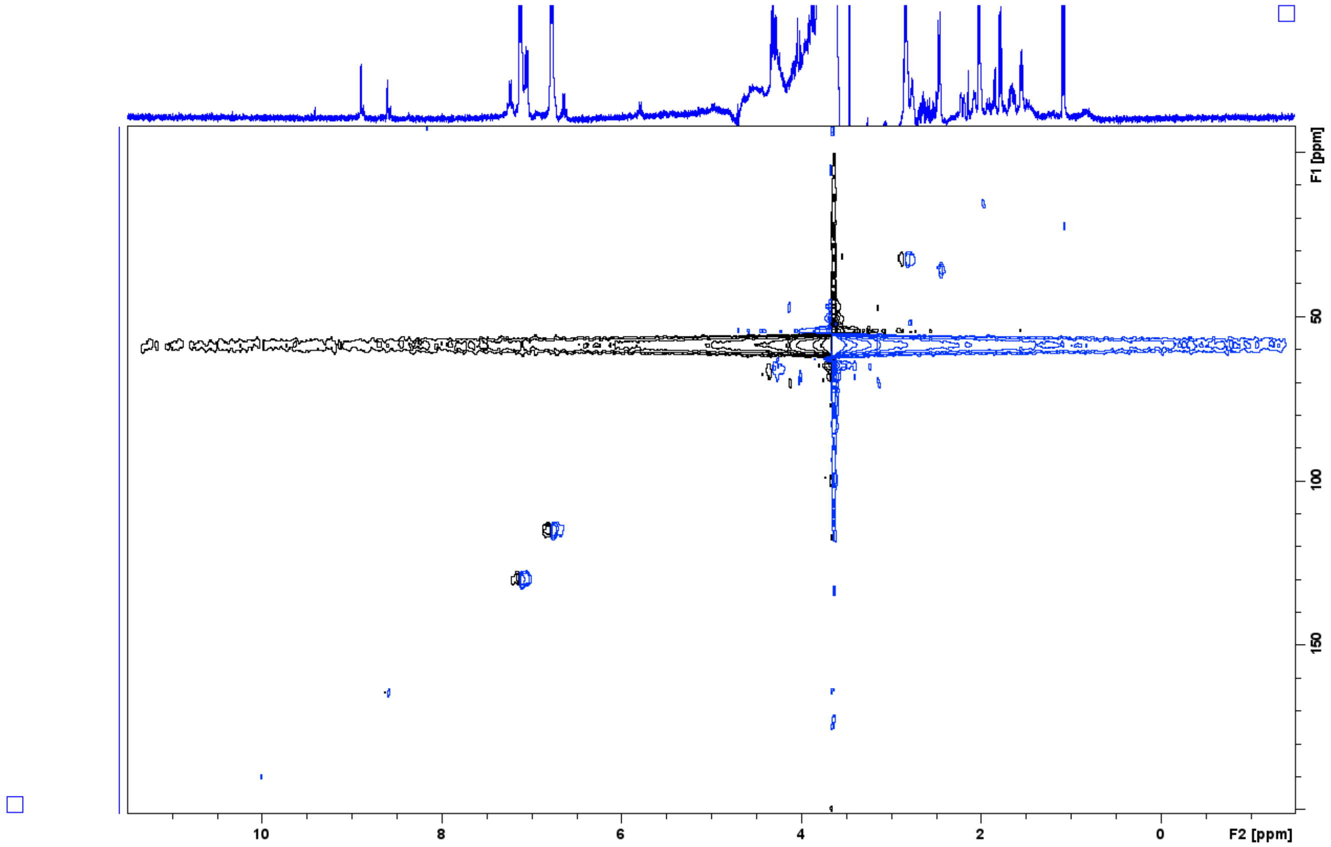

Figure S26 Zoom of HSQC of Oleocanthal and Tris HCl mixture in D_2O.

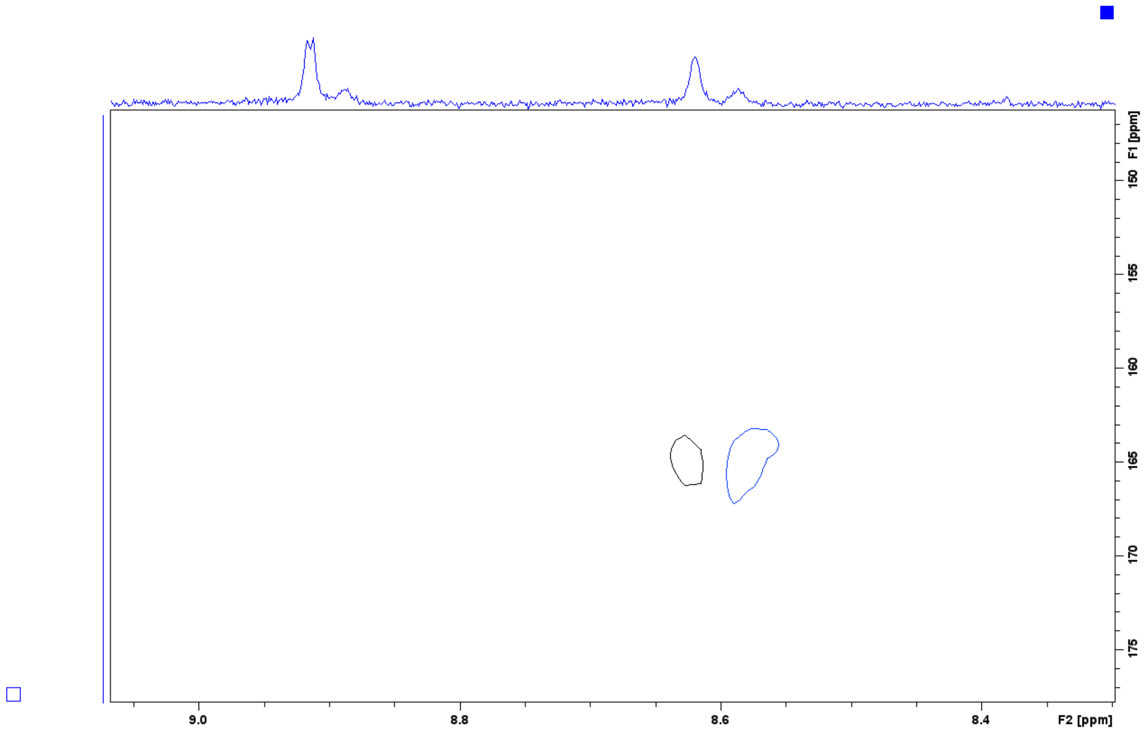

Figure S27 Zoom of HSQC of Oleocanthal and Tris HCl mixture in D_2O.

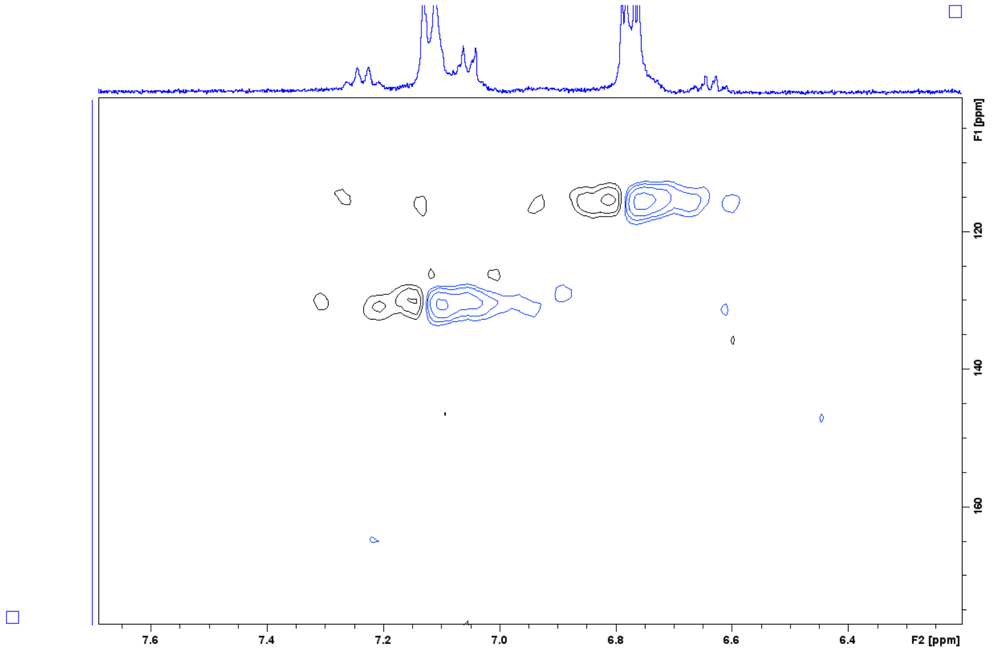

Figure S28 1D Selective Gradient TOCSY freq: 2.015ppm (400 MHz, D_2O) of Oleocanthal and Tris HCl after 300 min.

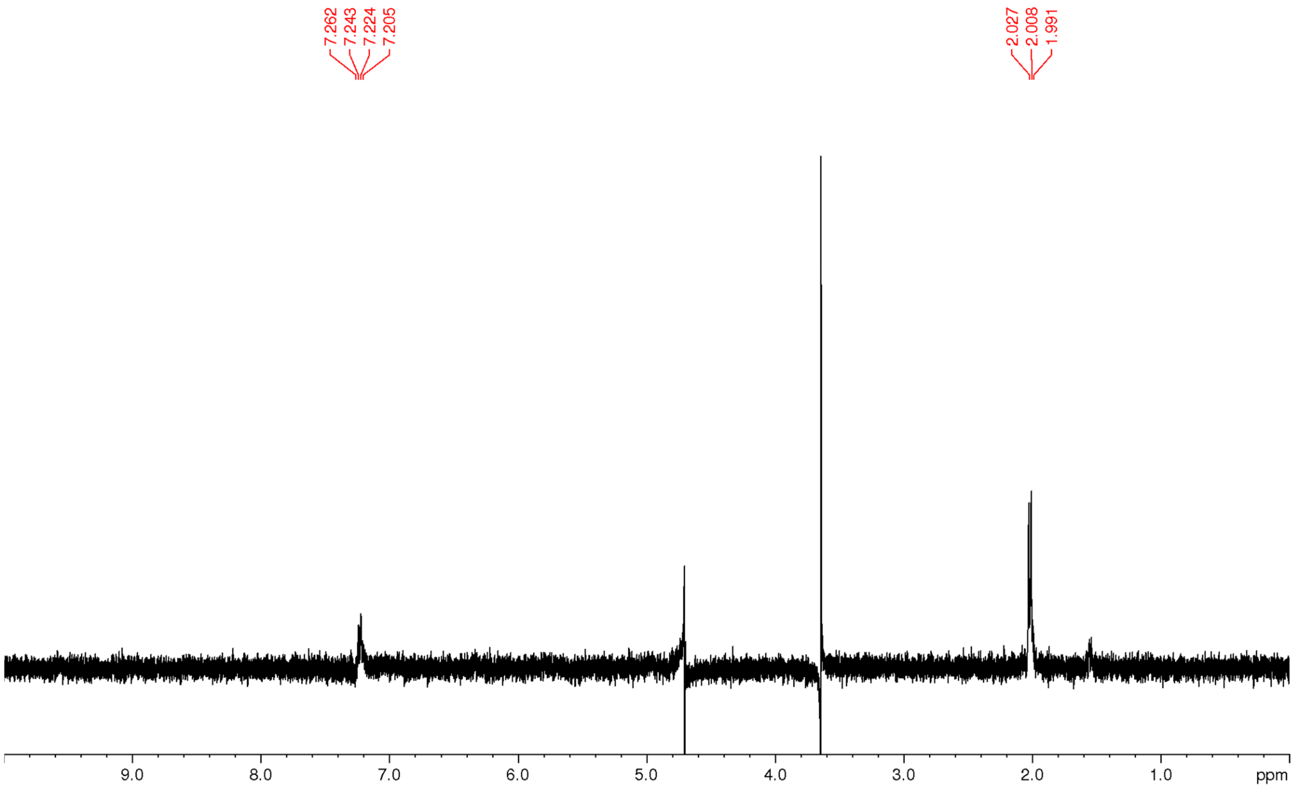

S5. NMR spectra of isolated adduct

Figure S29 ^{1}H NMR-spectrum (400 MHz, D_2O) of Oleocanthal adduct.

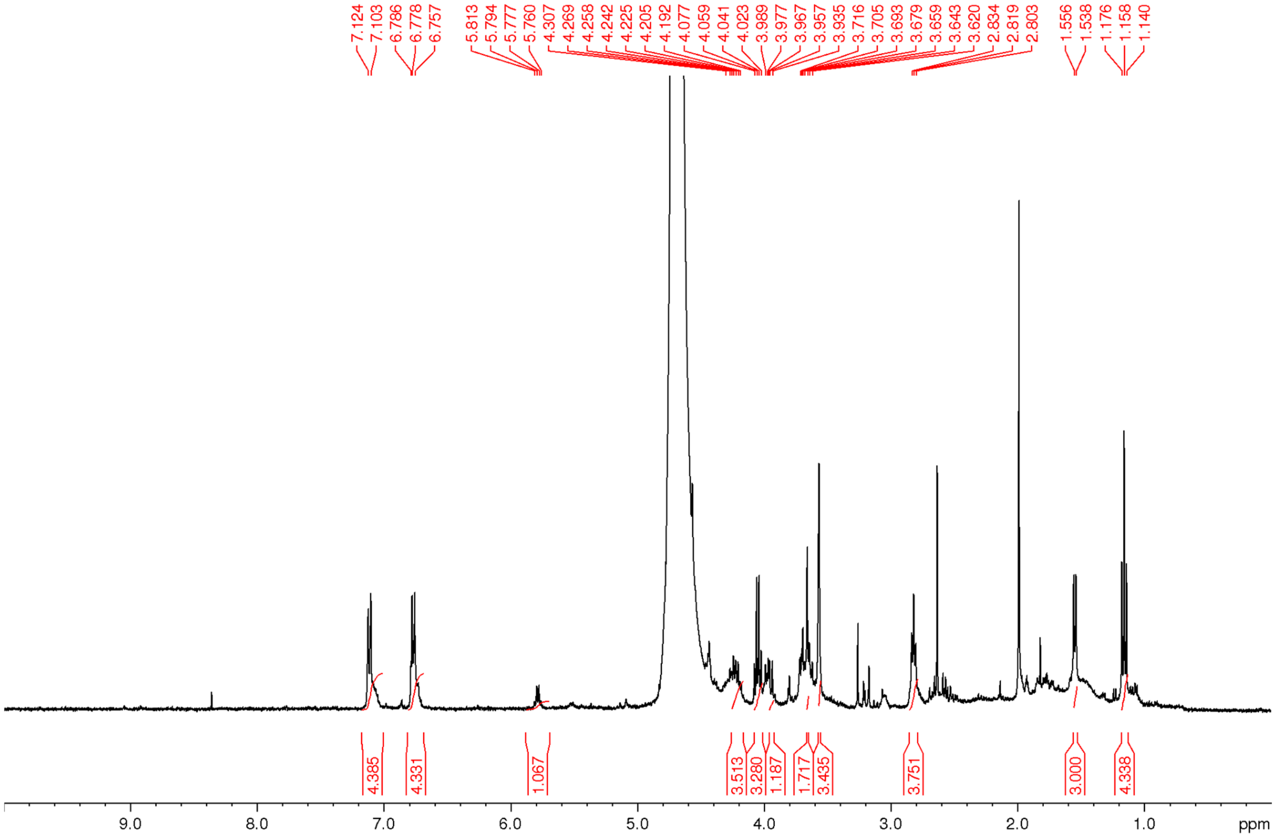

Figure S30 ^{1}H NMR-spectrum (400 MHz, D_2O) of Oleocanthal adduct signal zoomed view.

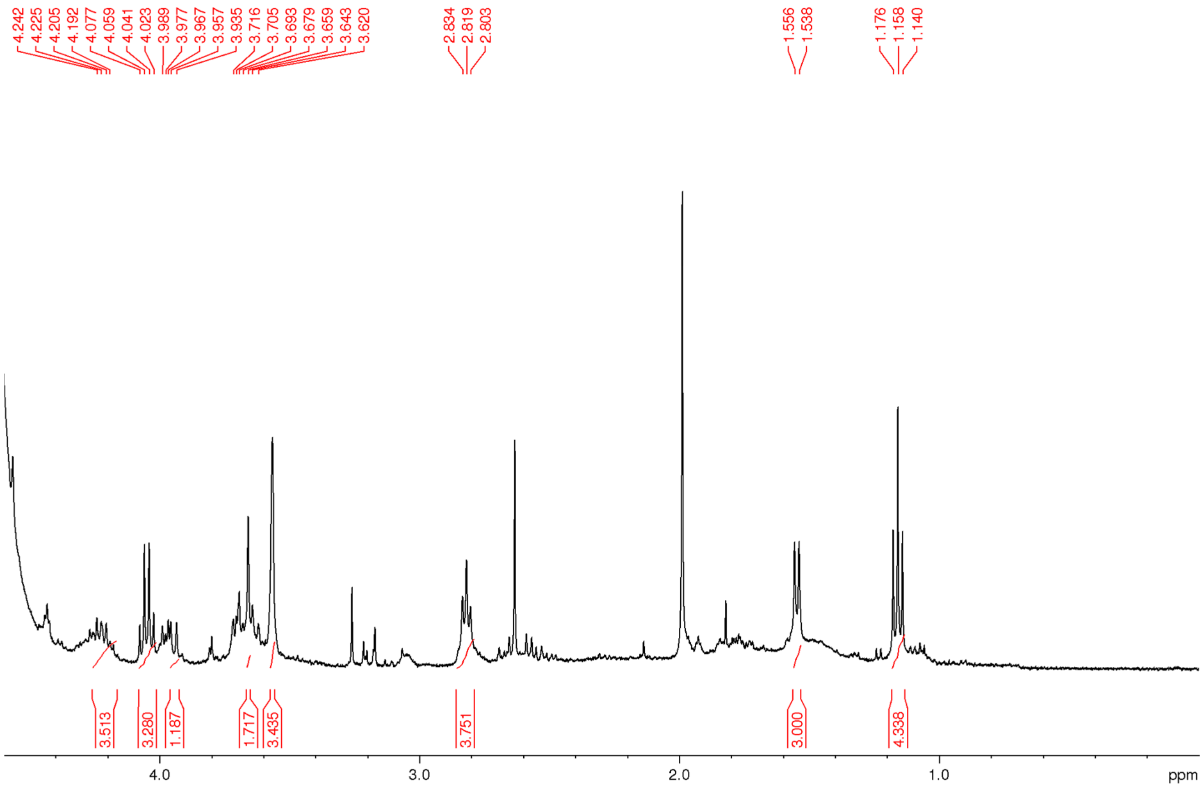

Figure S31 ^{1}H NMR-spectrum (400 MHz, D_2O) of Oleocanthal adduct with water suppression signal.

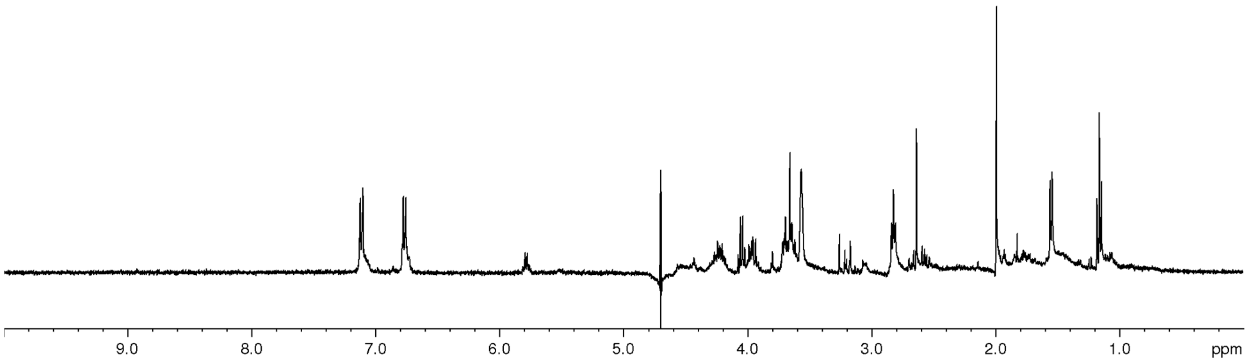

Figure S32 1D Selective Gradient TOCSY freq: 1.158pp NMR-spectrum (400 MHz, D_2O) of Oleocanthal adduct.

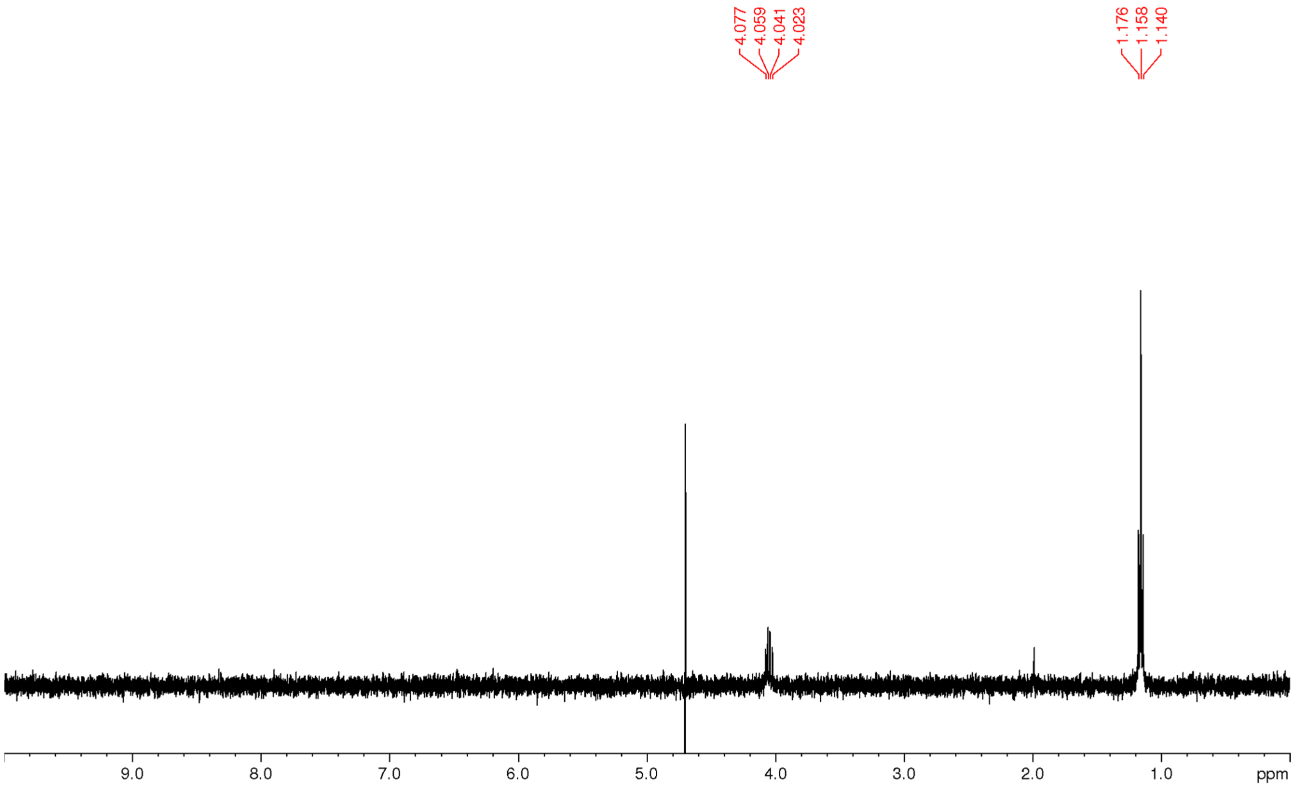
4.077
4.059
4.041
4.023
1.176
1.158
1.140
9.0
8.0
7.0
6.0
5.0
4.0
3.0
2.0
1.0
ppm

Figure S33 1D Selective Gradient TOCSY freq: 1.544ppm NMR-spectrum (400 MHz, D_2O) of Oleocanthal adduct.

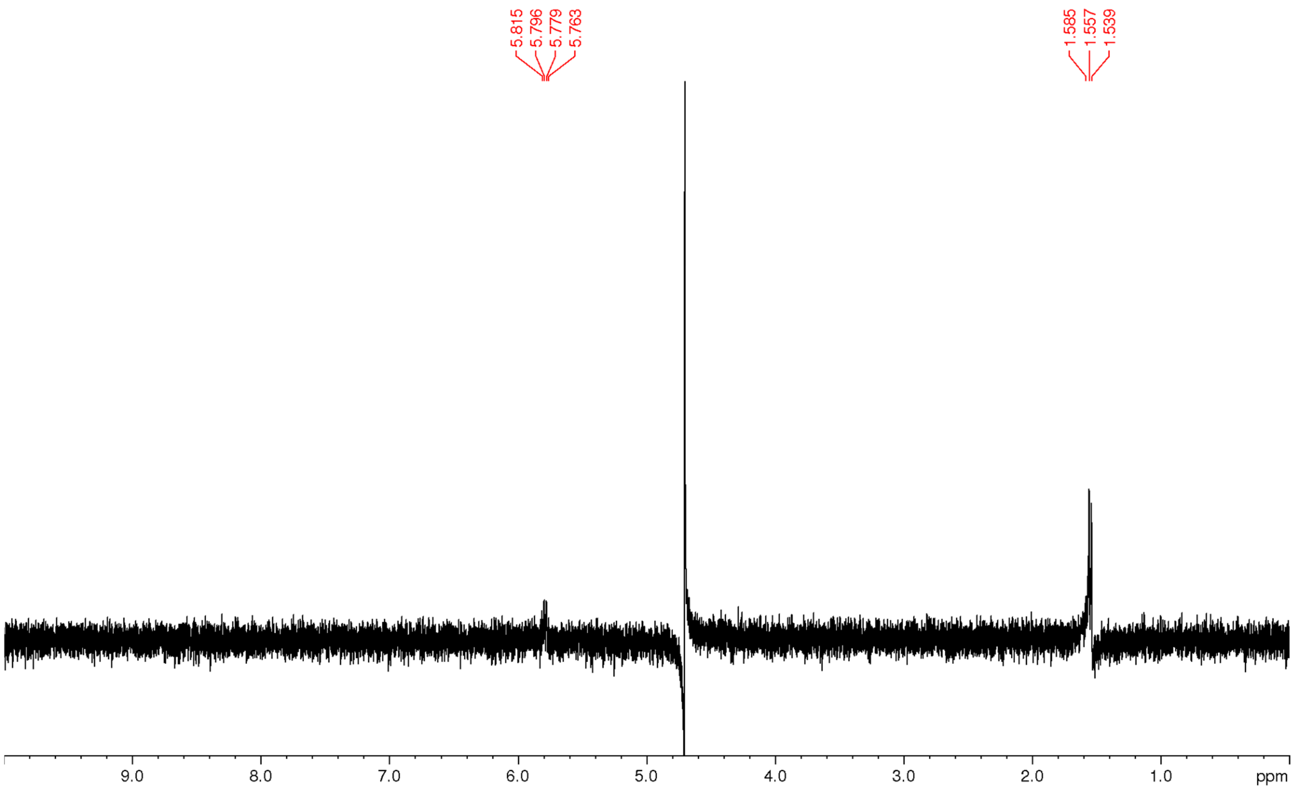

Figure S34 2D COSY of Oleocanthal adduct and Tris HCl mixture in D_2O.

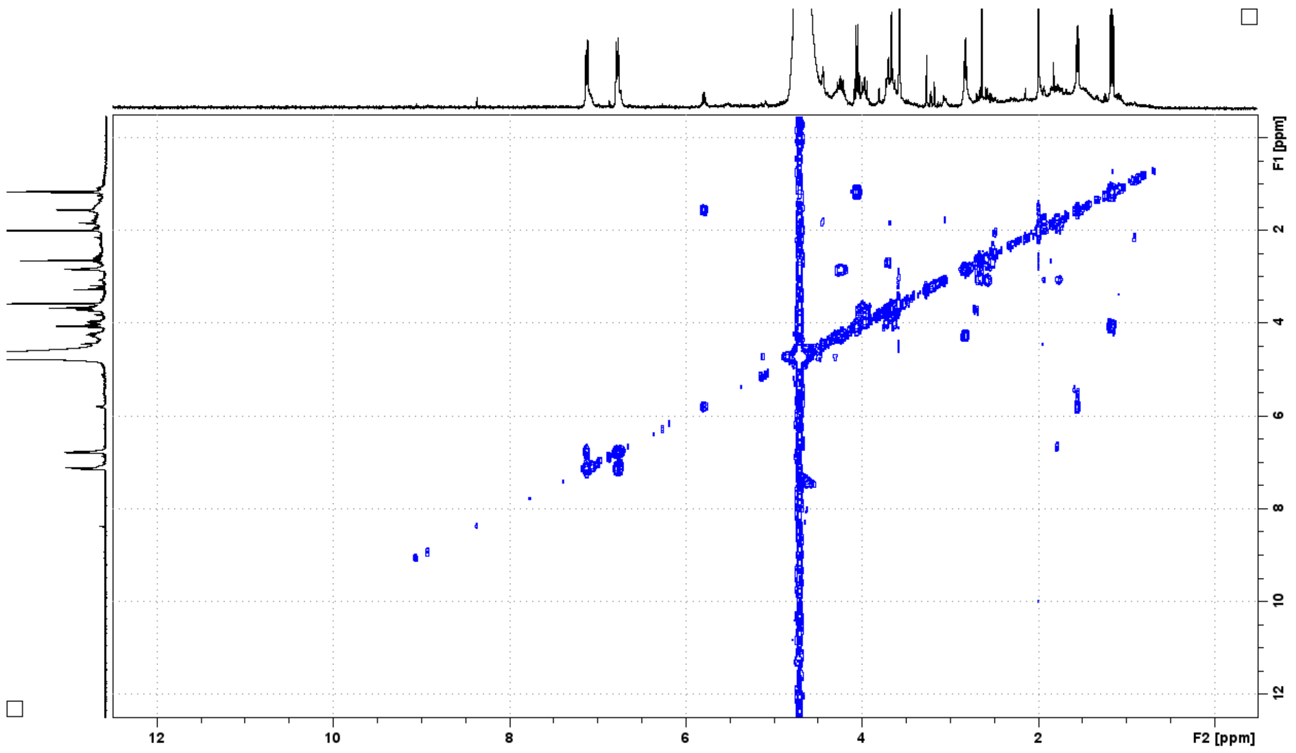

Figure S35 2D COSY of Oleocanthal adduct and Tris HCl mixture in D_2O: zoomed view.

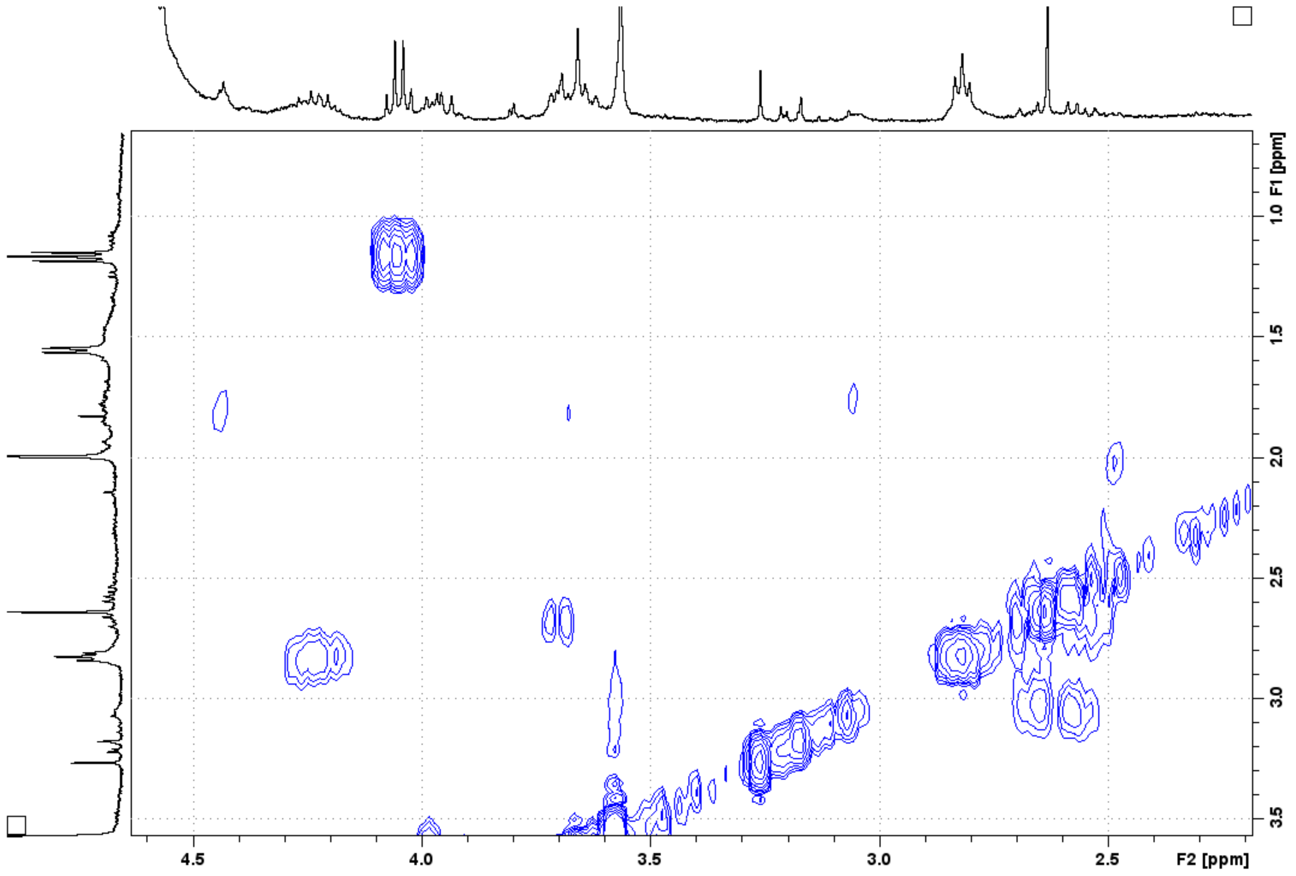

Figure S36 2D COSY of Oleocanthal adduct and Tris HCl mixture in D_2O: zoomed view.

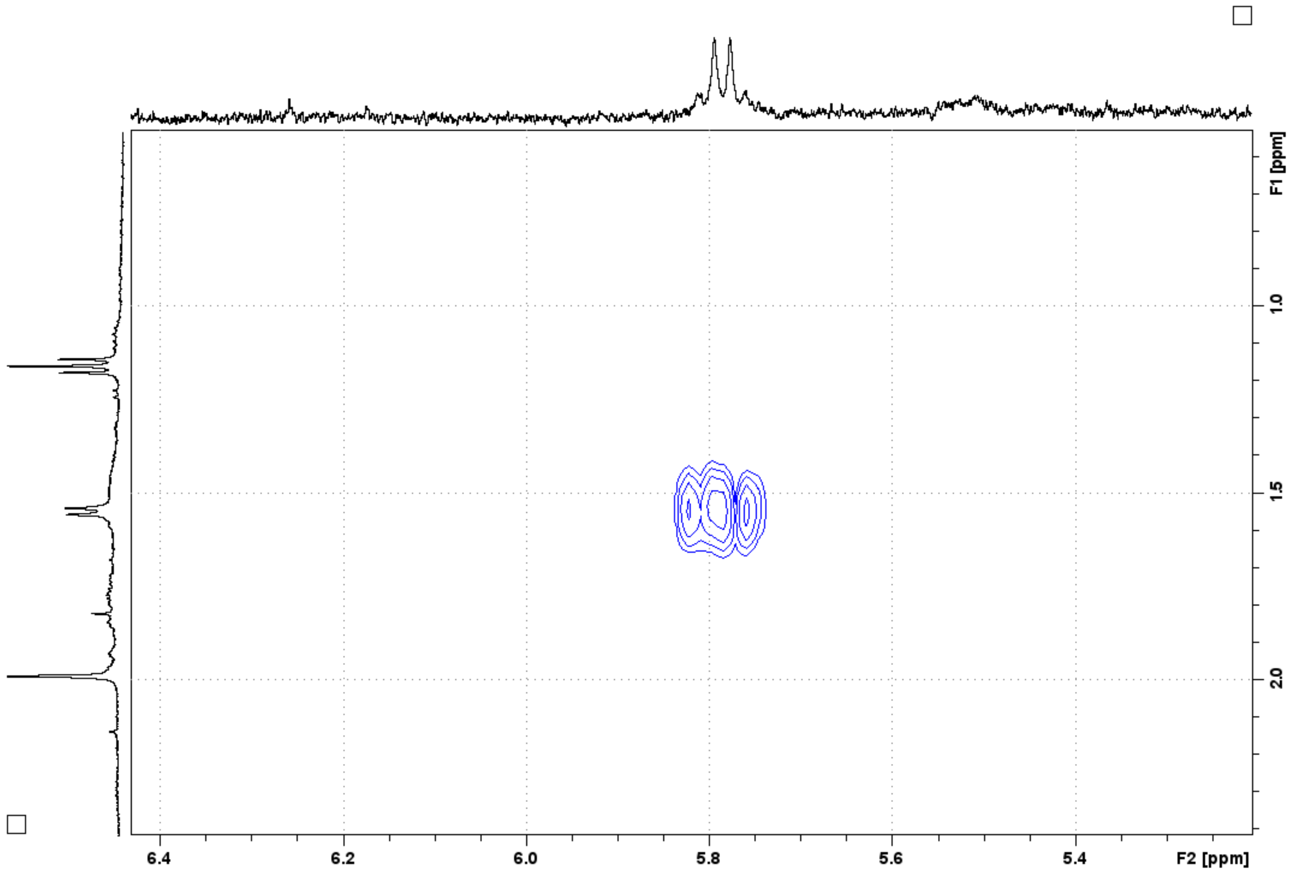

Figure S37 ^{1}H-NMR of Oleo, Oleo adducts and Oleo + Tris HCl mixture in D_2O.

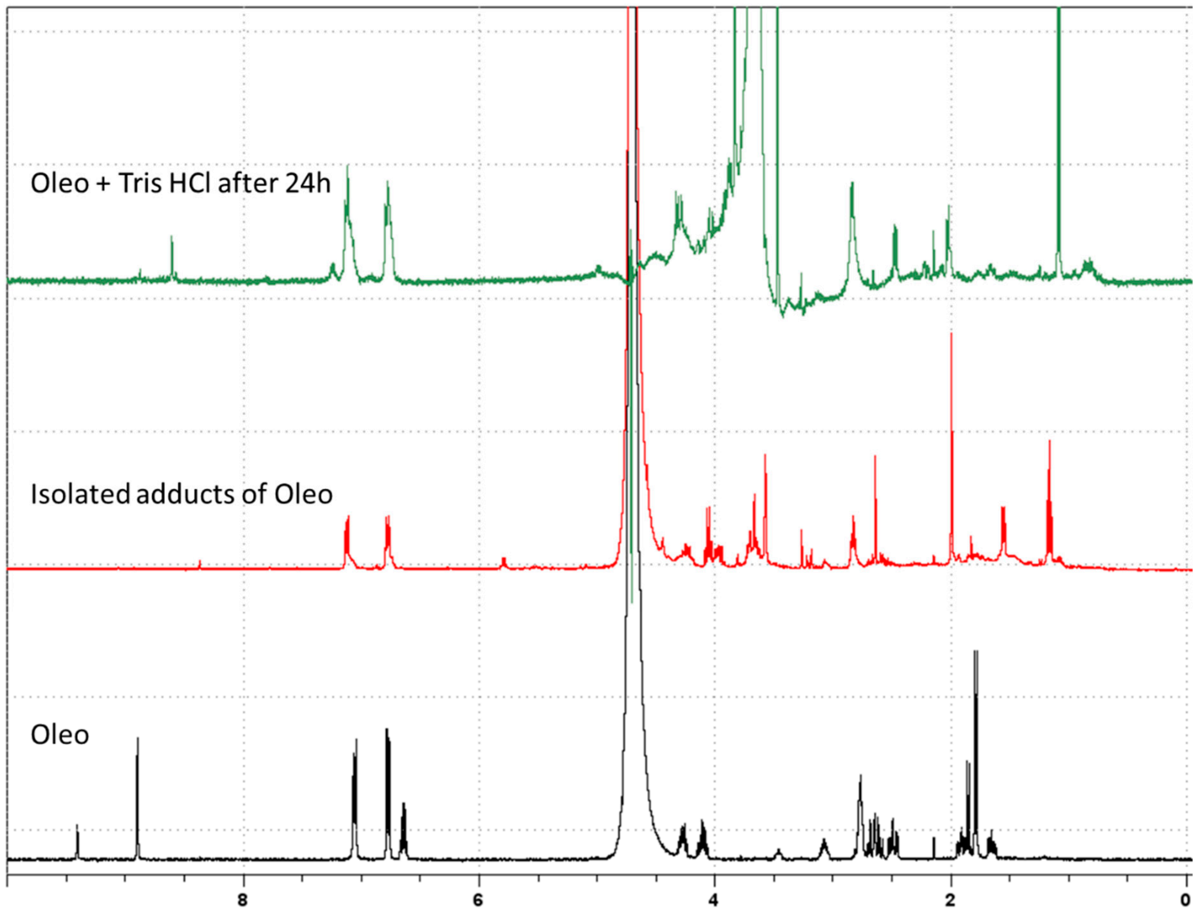

Figure S38 Mass spectra of Oleo-Tris, extracted from Total-Ion Current (TIC) trace, after the purification process.

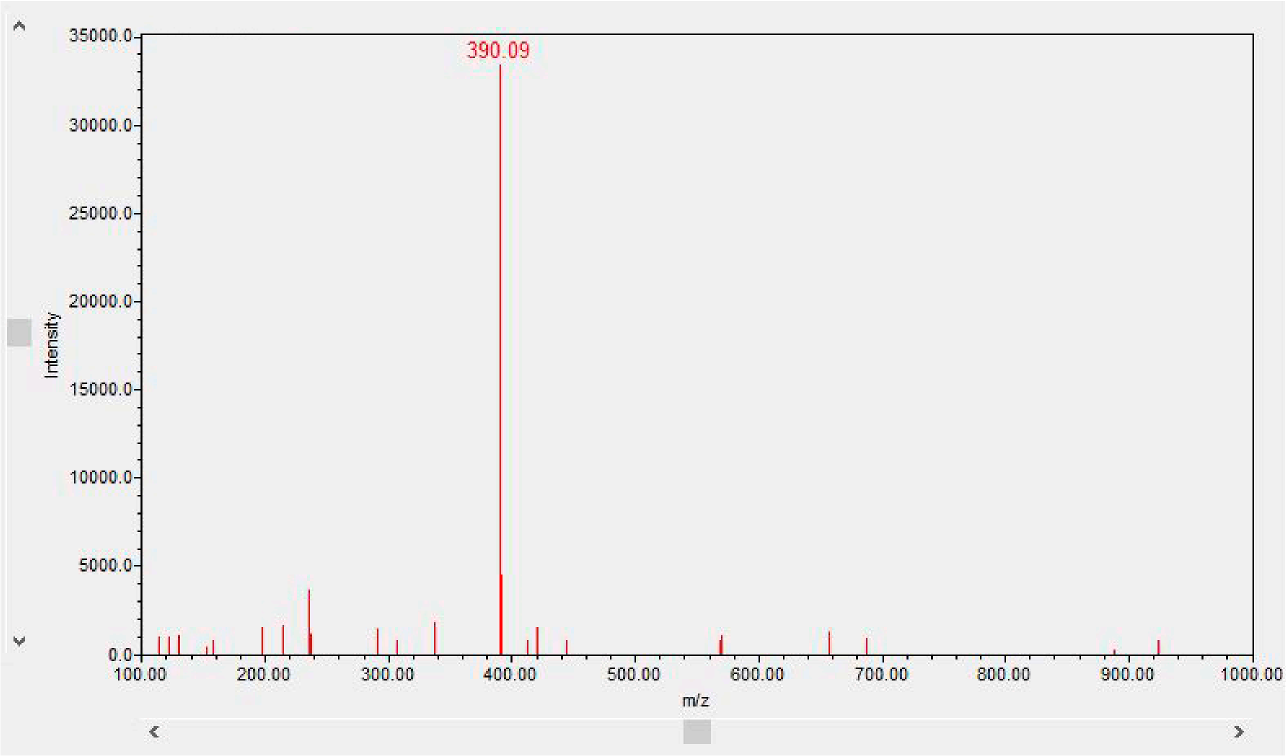

Supplement: Supplementary file 1 [file molecules-30-01645-s001.zip › molecules-3419236-supplementary.pdf]
